# Developing a Cell Quenching Method to Facilitate Single Cell Mass Spectrometry Metabolomics Studies

**DOI:** 10.1021/jacsau.5c00327

**Published:** 2025-05-06

**Authors:** Shakya Wije Munige, Deepti Bhusal, Zongkai Peng, Dan Chen, Zhibo Yang

**Affiliations:** † Department of Chemistry and Biochemistry, 6187 University of Oklahoma, Norman, Oklahoma 73019, United States; ‡ Stephenson Cancer Center, University of Oklahoma Health Sciences Center, Oklahoma City, Oklahoma 73104, United States

**Keywords:** Single-probe, single-cell mass spectrometry, cell quenching, freeze-drying, −80 °C storage, metabolomics

## Abstract

Single-cell mass spectrometry (SCMS) has emerged as a powerful tool for analyzing metabolites in individual cells, including live cells. However, cell metabolites have a rapid turnover rate, whereas maintaining metabolites’ profiles of live cells during sample transport, storage, or extended measurements can be challenging. In this study, a cell preparation method, which integrates cell washing by volatile salt solution, rapid liquid nitrogen (LN_2_) quenching, freeze-drying in vacuum, and freezer storage at −80 °C, to preserve cell metabolites for SCMS measurement is discussed. Experimental results revealed that LN_2_ quenching preserved the overall cell metabolome, whereas storage at −80 °C for 48 h slightly changed the metabolite profiles in quenched cells. However, metabolites in unquenched cells were changed regardless of low-temperature storage. The influence of omission of quenching and low-temperature storage on cell metabolites and relevant pathways were investigated. Results from this work indicate that cell quenching is necessary, but low-temperature storage time should be minimized to preserve cell metabolites. The method developed in the current work can be readily adopted by SCMS techniques with storage remaining largely unaltered, allowing for extended SCMS studies.

## Graphical abstract

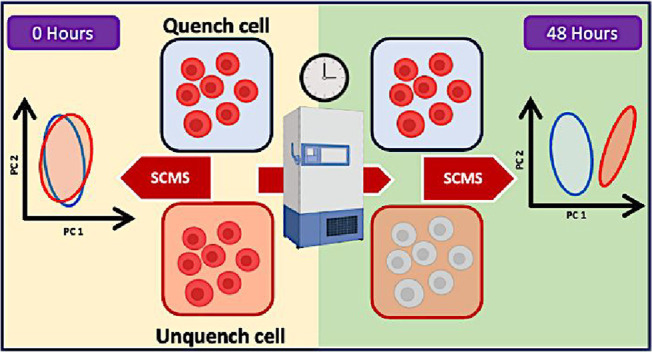

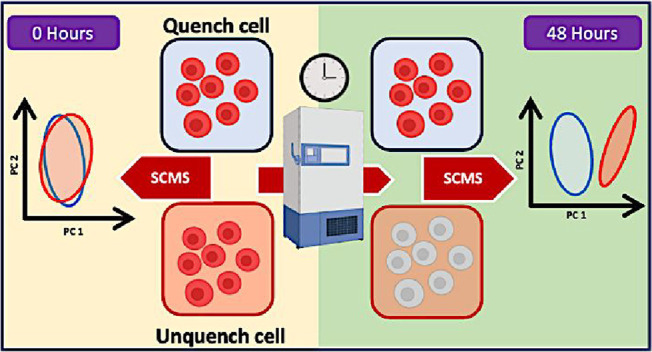

## Introduction

The ability to detect cell-to-cell variation allows for the discovery of hidden mechanisms that may be intractable to studies using bulk samples.^1^ Single-cell analysis has become a powerful tool in biological research, enabling a deeper understanding of the complexity and heterogeneity inherent in biological systems. This approach allows for studying unique characteristics, such as gene expression, protein levels, and metabolomic features, at the cellular level. Single-cell analysis enables us to identify rare cell populations and subpopulations with unique functions or characteristics. Single cell analysis has revolutionized research in numerous fields, opening new avenues for discovery and advancing our understanding of life at the single-cell level.^2^ Such analysis unveils crucial insights into multiple aspects, such as developmental processes, disease progression, and therapeutic responses, in studies of disease mechanisms and personalized treatment.

The area of single-cell analysis presents multiple challenges, including very limited sample amounts (e.g., the volume of a typical mammalian cell ranges between 1 and 10 pL)^3–5^ and extremely complex compositions (e.g., ∼2–4 million proteins/μm^3^ and >42,000 metabolites in a cell).^6,7^ Omics endeavors to thoroughly characterize all elements of cellular systems. Numerous cutting-edge technologies have been employed to study genomics,^8^ epigenomics,^9^ transcriptomics,^10^ proteomics,^11^ and metabolomics^12^ at the single-cell level. The metabolome, encompassing the entirety of a cell’s metabolites, emerges as a sensitive response to cell status and alterations in its surroundings. Unlike genes and proteins, which represent the cell’s potential capabilities, the metabolome has more rapid (e.g., within a few seconds) response to environmental perturbations.^13,14^ Studying the metabolome provides a unique lens into the immediate impact of environmental changes on the cell’s functional state, offering insights that extend beyond the capabilities of genomic and proteomic analyses. Thus, in addition to above-stated challenges (i.e., extremely limited sample amount and complex compositions), metabolomics studies of single cells, particularly for cells in their living status, need to minimize the influence of rapid turnover rates on profiles of cell metabolites during data acquisition.^15^

Multiple techniques, including nuclear magnetic resonance (NMR) spectroscopy, fluorescence microscopy,^16,17^ and mass spectrometry (MS), are commonly used for conventional metabolomics studies. Among them, MS-based methods are more effective for single cell metabolomic analysis due to its unique advantages: highly sensitive for detection and highly accurate for identification of extremely low abundance molecules with complex compositions.^2,18^ Several types of single-cell MS (SCMS) methods, categorized as either vacuum-based or ambient-based techniques, according to their sampling and ionization conditions, have been created and utilized for examining various cell types, including plant cells, mammalian cells, and yeasts.^15,19–22^ Vacuum-based SCMS methods predominantly rely on two approaches: secondary ion mass spectrometry (SIMS) and matrix-assisted laser desorption-ionization (MALDI) mass spectrometry. These techniques employ high-energy ion beams (for SIMS) or ultraviolet (UV) laser pulses (for MALDI-MS) to desorb and ionize cellular molecules, including metabolites, lipids, and pharmaceuticals, enabling sensitive and consistent analysis at the individual cell level.^19,23^ Unlike vacuum-based methods, ambient SCMS techniques enable analysis of cells with minimal or no sample preparation.^24,25^ Various ambient SCMS methods have been developed, including laser ablation electrospray ionization (LAESI) MS,^26^ live single-cell video-MS, induced nanoESI (InESI) MS,^27^ nanospray desorption electrospray ionization (nano-DESI) MS,^28^ probe electrospray ionization (PESI),^29,30^ and methods integrated with microfluidic chips^31–33^ and flow cytometry.^34^ We have developed the Single-probe, a multifunctional device that can be coupled to MS for single cell studies,^35–37^ MS imaging of tissues,^36,38^ and analysis of extracellular molecules within live spheroids^39^ in ambient environment. In addition, we have created the T-probe^40,41^ and micropipette capillary^42^ for SCMS measurements. These methodologies offer significant potential for exploring basic cell biology (e.g., cell heterogeneity,^43–45^ cell–cell interactions,^16^ and influence of environment on cell metabolism^46,47^ ) and potential clinical applications (e.g., quantification of drug^48^ and signaling molecules in single cells,^49,50^ drug resistance,^36,51,52^ and drug influence on cell metabolism^49,51,53^ ). Among them, the single-probe SCMS technique is routinely used for our SCMS metabolomics studies of live cells.

Although most ambient-based SCMS techniques allow for the analysis of live cells, they generally have relatively low throughput (e.g., 15 cells from nano-DESI MS,^28^ 32 cells from microprobe Capillary electrophoresis (CE)-ESI-MS,^54^ and ∼100 cells/day from Single-probe SCMS),^37^ largely due to necessary manual selection and analysis of individual cells. Because of the dynamic nature of the cell metabolism, cell metabolites may vary during a lengthy sample preparation and measurement. To preserve metabolomics features of live cells, researchers used quenching methods after cell isolation.^5,55^ Quenching can stop cellular metabolism and metabolomic transformations^56^ by lowing temperature^57^ (e.g., using liquid nitrogen (LN_2_) for snap freezing)^58,59^ or denaturing enzymes^60^ (e.g., adding organic solvents or acidic solutions)^58,61,62^ of cells. Quenching is pivotal to effectively arresting the cells’ metabolic activities, encapsulating a momentary freeze-frame of its biochemical state.^63^ This is crucial for accurate metabolomic studies in which capturing the precise temporal details of cellular metabolites is essential for understanding cellular function.

An effective protocol for quenching should take certain factors into consideration to achieve rapid and thorough inhibition of intracellular metabolic reactions.^59^ Studies have been performed to evaluate the performance of different quenching protocols, including cold isotonic saline (0.9% NaCl),^64^ chilled acetonitrile (at −40 °C),^61^ cold methanol (60%, at −40 °C) containing buffer salts (e.g., ammonium bicarbonate,^62^ NaCl,^59,62,64^ HEPES,^62,65^ ammonium carbonate^65^), ice-cold phosphate-buffered saline (PBS),^58,66^ LN_2_,^58,59,67^ and hot air treatments.^68^ In fact, some of these above quenching methods were designed for MS metabolomics studies of bulk cell samples,^59,61,64,66^ and cold methanol and acetonitrile have been utilized with Pico-ESI-MS^5^ and MALDI-MS techniques,^61^ respectively, for single cell studies. Although these quenching methods have demonstrated their efficacy in halting enzymatic activity and preserving cellular metabolites, each approach has its own limitations: organic solvents could lead to metabolite leakage and cell membrane damage;^64,69^ using solutions containing nonvolatile salts can severely impact MS analysis due to matrix effect,^61^ which leads to ion suppression,^70^ reduced sensitivity, inaccurate quantification of analytes,^62,71^ and ion signal interference.

LN_2_ snap freezing has been widely used in biological research.^72^ Instead of using cold organic solvents containing buffer salts, quenching by LN_2_ seems more suitable for SCMS studies because LN_2_ can immediately stop metabolomic activities without leaving residual nonvolatile salts after LN_2_ evaporation. However, previous studies showed LN_2_ snap freezing often led to cell membrane damage,^73,74^ which is undesirable for SCMS studies. To prevent cell membrane damage in LN_2_ quenching, a method combining fast filtration, NaCl solution washing, and LN_2_ freezing was employed for the metabolome analysis of suspended animal cells.^63,69^ Briefly, cell suspension was quickly filtered using glass fiber filter disk under vacuum, and the filter containing cells was rinsed by cold iso-osmotic NaCl solution to remove residual culture medium and then frozen in LN_2_. This method is effective to retain metabolites, including those with high turnover rates, and mitigate cell membrane damage,^63,69^ but it is unlikely suitable for SCMS studies because of challenges to isolate cells for experiment and matrix effect due to remaining nonvolatile salts. In addition to LN_2_ quenching, sample storage in a −80 °C freezer is commonly used to preserve cells and tissues prior to analysis. However, the influence of storage at −80 °C on the metabolite profiles of single cells has not been previously reported. There is a crucial need for developing new cell quenching methods for robust SCMS metabolomics studies.

In the current work, we developed a new protocol that combines cell washing by volatile salt solution, LN_2_ quenching, freeze-drying in vacuum, and low-temperature storage, for sensitive ambient SCMS analysis. An advantage of our method is to incorporate a rapid washing^67^ utilizing the solution containing ammonium formate (AF), which is compatible with live cells and MS analysis,^5^ prior to rapid LN_2_ quenching to minimize cell membrane damage. Quenched cells are then rapidly dried in a vacuum with the presence of residual LN_2_ to efficiently remove water molecules from cells, allowing for minimized metabolic activities and degradation of metabolites of cells during SCMS measurement in an ambient environment. We also evaluated the influence of storage in a −80 °C freezer on cells’ metabolites. Our methods can be readily adopted by researchers for robust SCMS metabolomics studies using other types of techniques.

## Experimental Section

### Cell Culture

HCT-116 and HEK-293T cells were grown in McCoy’s 5A and DMEM media, respectively. Both cell culture media (Fisher Scientific Company LLC, IL, USA) were supplemented with 10% fetal bovine serum (FBS, GE Healthcare Bioscience Corp, Marlborough, MA, USA) and 1% penicillin-streptomycin (Life Technologies Corporation, Grand Island, NY, USA). Cells were cultured in an incubator (HeraCell, Heraeus, Germany) at 37 °C in the presence of 5% CO_2_. Cells were passaged every 2 days when their confluence reached 80%. To perform cell passaging, 2 mL of trypsin-EDTA (Life Technologies Corporation, Grand Island, NY, USA) was introduced into a Petri dish and incubated at 37 °C for 3 min to detach the cells. Following this, 8 mL of cell culture medium was added to deactivate the trypsin enzymatic activity. Subculturing was carried out by transferring 1 mL of the cell suspension solution into 9 mL of a fresh culture medium.

Cell seeding was performed using cell suspension solution (∼1 × 10^6^ cells/mL) in a culture medium. Four glass coverslips (18 mm, VMR micro cover glass, USA. CAT. No. 48380046) were individually placed in four wells of a 12-well plate. An aliquot of 2 mL/well of cell culture media was transferred to these four wells, and 200 μL (∼2 × 10^5^ cells/well) of cell suspension solution was added into each well containing a coverslip. The prepared 12-well plate was kept in the incubator overnight, allowing cells to attach onto glass coverslips.

### Cell Washing, Quenching, Drying, and Storage

A series of experiments with different procedures were performed to prepare cells, including washing, quenching, freeze-drying, and storage, to evaluate their influences on cell metabolomics profiles (Figure 1).

**Figure 1:**
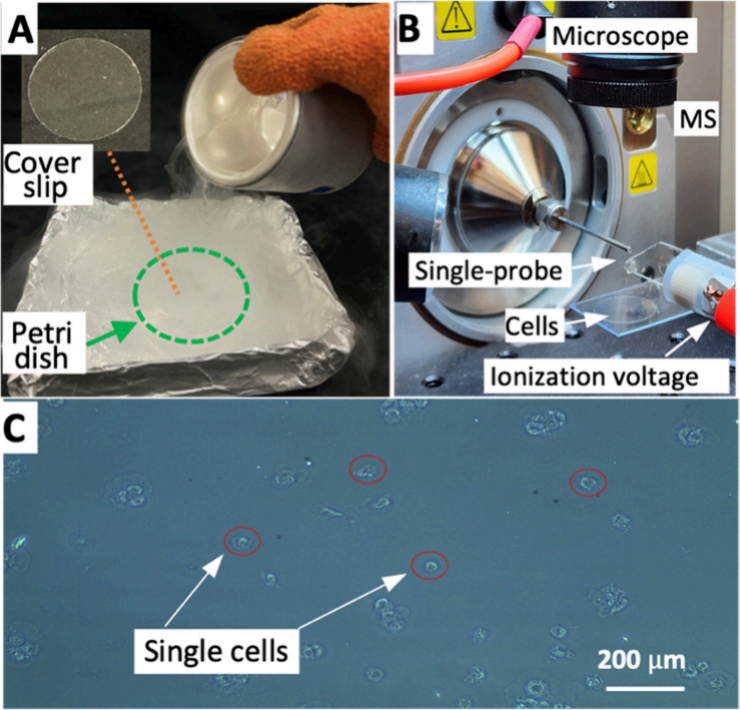
Cell quenching and SCMS setup. (A) Cell quenching by LN_2_. The inset shows the cell containing glass coverslip in a Petri dish. (B) SCMS setup. (C) Photo of HCT-116 cells after quenching.

### Cell Washing by Cold Ammonium Formate (AF) Solution

It has been reported that AF solution (0.1427 M or 0.9%) is compatible with live cells and has a minimum influence on cell metabolism.^5^ This washing step can replace nonvolatile salts (e.g., Na^+^, K^+^, and Mg^2+^ in culture medium) with volatile AF, significantly reducing the matrix effect in SCMS experiments while minimizing alterations of metabolites in live cells. To perform cell washing, 0.9% AF (w/w, 0.45 g in 50 mL of Optima LC/MS-grade water (Fisher Chemical, USA)) was prepared and stored at 4 °C. Next, 2 mL of cold AF solution was added into each empty well in a 12-well plate. Last, each coverslip containing cells was rapidly rinsed in a well containing AF solutions, and this washing step was repeated for the second time.

### Cell Quenching by LN_2_

Rinsed coverslips containing cells were placed in an open Petri dish, which was placed into a container (e.g., folded by aluminum foil). 10–20 mL of LN_2_ was carefully poured all over the Petri dish containing coverslips to ensure rapid freezing (3 to 5 s). Excessive LN_2_ was cautiously removed by tilting the Petri dish with a tweezer. This step must be carried out quickly to prevent formation of large ice crystals due to residual moisture from the earlier washing step.

### Cell Freeze-Drying in Vacuum

Quenching was used to stop enzymatic activity at ultralow temperatures, whereas drying at a low temperature removed water molecules from cells to deactivate enzymes during SCMS measurements under ambient conditions. Freeze-drying was performed by placing cold Petri dish (with residual LN_2_) containing coverslips into a SpeedVac (Thermo Scientific, Savant SPD111 V) performed under its standard vacuum condition. The rotor of the SpeedVac was removed to accommodate the Petri dish. Cell drying can be accomplished within 5–7 min following the standard drying procedures.

### Cell Storage in a −80 °C Freezer

To test the influence of low temperature storage on the cell’s metabolomics profiles, dried cells were stored in a −80 °C freezer, aiming to minimize changes in cellular metabolites. After 48 h of storage, dried cells were taken out from the −80 °C freezer and then immediately placed into a desiccator (at room temperature) to eliminate water condensation. Cells were maintained in the desiccator for ∼10 min, allowing them to reach room temperature prior to SCMS experiments. Cells preserved their integrity after LN_2_ quenching, freeze-drying, and storage in −80 °C freezer (Figure S1).

To evaluate the influence of quenching and storage on cell metabolites, we prepared cells using different protocols and performed SCMS experiments (Figure S2A). Four groups of cells (i.e., Groups 1, 2, 3, and 4) were prepared using different processes (Table 1, Figure S2B). Cells in all groups were washed with AF solution before undergoing additional processes.

**Table 1:** Cell Groups Prepared Using Different Processes for the Single-Probe SCMS Measurements^a^

	Cell preparation procedures		
Cell groups	Quenching	Drying	Storage
1	Yes	Freeze	No
2	No	RT	No
3	Yes	Freeze	Yes (−80 °C, 48 h)
4	No	RT	Yes (−80 °C, 48 h)

a Cells were washed using AF solution (0.9%) prior to sequential processing. Washed cells were subjected to LN_2_ quenching (Groups 2 and 4) or no quenching (Groups 1 and 3), dried (freeze-drying or at room temperature (RT)) in a vacuum (SpeedVac) and analyzed without storage or after storage at −80 °C (48 h).

#### Group 1

Cells in Group 1 underwent quenching and drying (no storage) prior to SCMS measurements. Cells in this group were served as the baseline control.

#### Group 2

To elucidate changes of cellular metabolites due to the omission of quenching, Group 2 represents freshly dried cells. Cells were dried under vacuum at room temperature and subjected to SCMS analysis without low temperature storage.

#### Group 3

To determine if storage at low temperature can preserve cell metabolites, cells in Group 3 underwent quenching, drying, and storage (at −80 °C for 48 h).

#### Group 4

To elucidate if storage at low temperature can preserve metabolites in freshly dried cells (no LN_2_ quenching), cells in Group 4 underwent drying and storage (at −80 °C for 48 h).

All four categories of cells were analyzed using the Single-probe SCMS method. ∼ 30 cells in each group were analyzed in both positive and negative ion modes, and ∼ 240 cells in total were measured. To minimize potential batch effects, glass coverslips containing cells from these four groups were placed on the XYZ-stage and cells were randomly selected for measurements. Analyses of all cells in the same ion mode were accomplished within the same day using the same experimental conditions (i. e., the same Single-probe device, solvent flow rate, and ionization voltage) by the same people.

### Single-Probe Fabrication and SCMS Setup

The Single-probe was fabricated in accordance with established procedures.^35^ A Single-probe comprises three primary components: a nanoelectrospray ionization (nano-ESI) emitter, a dual-bore quartz needle, and a fused silica capillary (Figure 2). The dual-bore quartz tubing (outer diameter 500 μm; inner diameter 127 μm, sourced from Friedrich & Dimmock, Millville, NJ) was pulled into sharp needles (tip size is ∼ 10 μm) using a laser-based micropipette puller (Sutter P-2000, Sutter Instrument, Novato, CA). The nano-ESI emitter was pulled while heating a fused silica capillary (outer diameter 105 μm; inner diameter 40 μm; Polymicro Technologies, Phoenix, AZ) with a butane micro torch. The assembly of a Single-probe entails inserting the fused silica capillary and nano-ESI emitter into the dual-bore quartz needle. To facilitate experimentation, the Single-probe was affixed to a microscope glass slide using epoxy adhesive. Subsequently, the Single-probe was mounted on an XYZ-stage system, and digital microscope (Shenzhen D&F Co., China) was used to monitor cells during the experiment. The entire setup was coupled with an Orbitrap Exploris 240 mass spectrometer (Thermo Scientific, Waltham, MA, USA) for the analysis of SCMS (Figure S3). Acetonitrile (with 0.1% formic acid) served as the solvent for the SCMS experiments at a flow rate of 150 nL/min. The mass spectrometer was configured with mass ranges of *m*/*z* 200–1500 in positive ion mode and *m*/*z* 70–900 in negative ion mode. Additional mass spectrometer settings include a mass resolution of 120 K (at *m*/*z* 200), an ionization voltage of 2.9 kV in positive mode and – 2.1 kV in negative ion mode, one microscan, a maximum injection time of 100 ms, and the use of an automatic gain control (AGC) Standard. MS/MS analysis of ions with relatively high abundances were conducted at the single-cell level using the following parameters: HCD mode, 150 nL/min flow rate, mass resolution of 120 K (at *m*/*z* 200), ionization voltage of 2.9 kV in positive ion mode and – 2.1 kV in negative ion mode, one microscan, a maximum injection time of 100 ms, and collision energy ranged between 10 and 35 NCE (Normalized Collision Energy) as shown in Figures S4 and S5.

**Figure 2:**
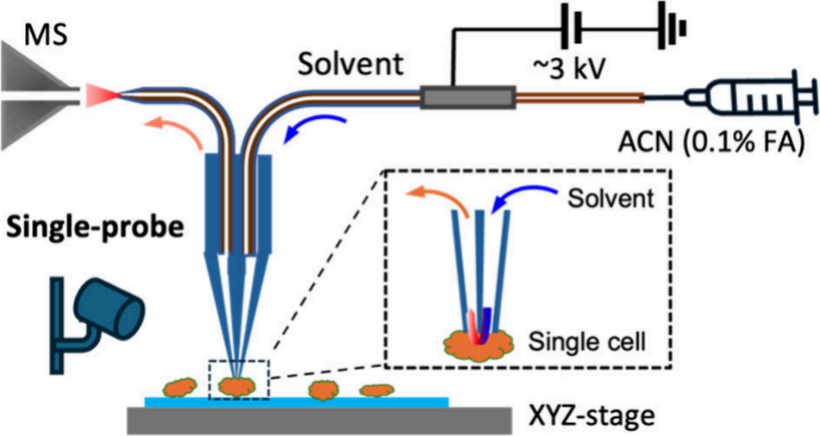
Single-probe SCMS setup for the analysis.

### Data Analysis

The raw SCMS data were subjected to pretreatment using a customized R script reported in our previous studies.^46^ The data pretreatment includes background removal (to remove signals originating from solvents and cell culture media), noise reduction (to remove instrument noise), and ion intensity normalization (to normalize the intensity of each ion to the total ion current (TIC)). Deisotope analysis was performed with Python package ms_deisotope v0.0.053 (mobiusklein.github.io/ms_deisotope). After the deisotope, peak alignment was performed using an in-house Python script. To extract essential biological information and perform comparison of metabolomic profiles among different groups of cells, pretreated SCMS data were processed for visualization (by Principal Component Analysis (PCA), heat map, and volcano plot), Random Forest analysis, and pathway analysis using MetaboAnalyst 6.0.^75^ PCA was used for dimensionality reduction and visualization of SCMS data, allowing for intuitive comparison of the overall metabolites’ profiles of cells from multiple groups. Heat maps were generated to visualize the relative abundances of metabolites among cells. The volcano plot was used to illustrate significantly changed (*p* < 0.05 from *t* test, FC > 1.5) species of cells in two different groups. Pathway analysis was employed to determine which metabolomic pathway significantly altered (FDR < 0.05) in pairwise comparison of cells in two groups. Pathway analysis examined the correlation between p-values (from pathway enrichment analysis) and pathway impact scores (from pathway topology analysis mapped against KEGG using *Homo sapiens* as the model organism). This comprehensive approach allowed for gaining deeper insights into the nuanced variations within the metabolic landscapes of the studied cell groups. Ions of interest were tentatively labeled by searching their accurate *m*/*z* values against both Human Metabolome Database (HMDB)^76^ and Lipid Maps.^77^ More confident molecular identification was carried out by comparing MS/MS spectra with those (experimental or in silico results) from these two databases or previously published data (Table S1 and Figure S6 and Figure S7). *Post hoc* power analysis (Table SI_1­(xlsx), Table SI_2 (xlsx)) of the SCMS data was carried out using an in-house Python script.

## Results and Discussion

Due to the rapid turnover rates of metabolites and relatively low throughput of most ambient SCMS techniques, cell metabolites may change during extended measurements. To overcome these challenges, we developed a method that integrates cell quenching, drying, and storage to preserve cell metabolites for ambient SCMS metabolomics studies. Cells processed under different conditions were analyzed using the Single-probe SCMS technique to evaluate the influence of experimental protocols on cell metabolites.

### PCA Illustrating the Influence of Sample Preparation on Overall Metabolites’ Profiles in Single Cells

To visualize the overall profiles of metabolites in individual cells across four different groups, PCA was carried out to analyze the SCMS data collected in both positive (Figure 3A) and negative (Figure 3B) ion modes.

**Figure 3:**
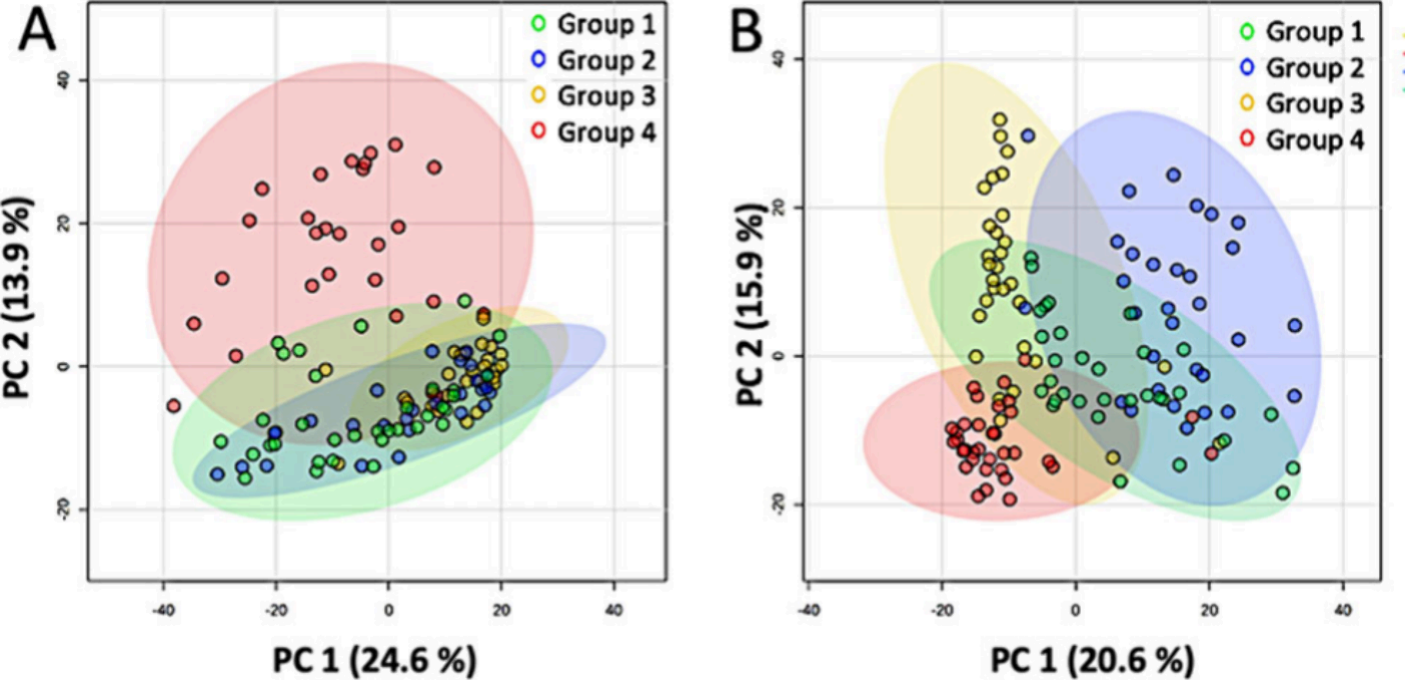
PCA of SCMS results obtained from HCT-116 cells in all four groups in the (A) positive and (B) negative ion modes.

#### Positive Ion Mode Results

A general trend can be observed: cells in Groups 1, 2, and 3 possess similar profiles of metabolites, whereas those in Group 4 largely distinguish them from the other three groups. Two major conclusions can be drawn from these results. First, quenching and low temperature storage largely preserved metabolites in dried cells. As illustrated in Figure 3A, the overall metabolite profiles between Group 1 (cells were freshly quenched and dried) and Group 3 (cells were quenched, dried, and stored at −80 °C for 48 h) are nearly indistinguishable. Storage at −80 °C is an effective way to preserve metabolites in dried cells. Second, freshly dried cells generally retained cell metabolites. The overall metabolomic profiles of cells in Groups 1 and 2 (cells were dried without quenching) are largely indistinguishable. These results indicate that rapid vacuum drying at room temperature generally preserved cell metabolites when cells were analyzed soon (e.g., within 30 min) after drying. However, the metabolites in unquenched cells changed after storage. Obvious difference of overall profiles of cell metabolites can be observed when comparing the results between Group 2 and Group 4 (unquenched cells, dried, and stored at −80 °C). Similarly, significantly different metabolomics profiles can be observed between Groups 3 and Group 4. Trends observed in PCA plots are also reflected in results obtained from Random Forest analysis (a higher classification error indicates a lower degree of distinguishability among groups). The classification error obtained from Group 4 (0.16) is lower than those from Groups 1 (0.27), 2 (0.44), and 3 (0.33), indicating that metabolites’ profile in Group 4 is more different from the other three groups sharing more similarities (Table S2).

#### Negative Ion Mode Results

Compared with results from the positive ion mode, metabolites’ profiles of cells from Groups 1, 2, and 3 obtained in the negative ion mode seem to have lower degrees of overlap (Figure 3B). The variation in clustering patterns could be attributed to different detection sensitivities between these two ion modes: certain molecules (e.g., PCs) are preferentially produced as cations, whereas others (e.g., fatty acids) are more efficiently generated as anions. Different types of cell metabolites could be affected by quenching and storage conditions to different degrees. If the key differentiating metabolites are more efficiently ionized in one ionization mode, then a clearer separation can be observed in the corresponding PCA plot. This observation can be further verified from differences in significantly altered metabolites (Table S1) and metabolomic pathways (Tables S3–S10) measured in two ion modes. Nevertheless, the same trend was observed from Random Forest analysis: the classification error obtained from Group 4 (0.03) is lower than those from Groups 1 (0.25), 2 (0.29), and 3 (0.17) (Table S11). Thus, results from both ion modes indicate that quenching is indispensable to preserve cellular metabolomic integrity, even for cells to be stored under a low temperature such as −80 °C.

### Heat Map Illustrating the Influence of Sample Preparation on Metabolites’ Relative Abundances in Single Cells

Heat maps were generated using SCMS data obtained from all four cell groups (Figure 4), depicting the changes in the abundances of the top 100 metabolites in both positive (Figure 4A) and negative (Figure 4B) ionization modes. The rows represent different metabolites, and the columns represent individual cells, with colors indicating the relative abundance of each metabolite. Clear trends can be observed for metabolites across 120 single cells in four groups (with 30 single cells in each group).

**Figure 4:**
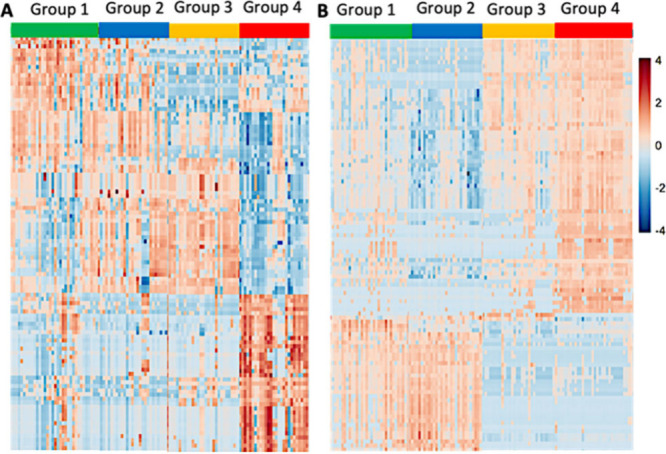
Heat maps summarizing metabolites measured in single HCT-116 cells under different preparation conditions. Relative abundances of top 100 metabolites in (A) positive and (B) negative ion modes.

#### Positive Ion Mode Results

Notably, the heat map revealed patterns among cells in different groups. The positive ion mode results indicate that cellular metabolites in Groups 1 and 2 share higher similarities, whereas some minor changes can be observed in Group 3. In contrast, cells in Group 4 exhibit drastically different trends compared to the other three groups.

#### Negative Ion Mode Results

Although cellular metabolites in Groups 1 and 2 possess similar patterns, which are also observed in the positive ion mode results, obvious changes can be seen in Groups 3 and 4. Apparently, cells in Groups 1 and 2 show higher similarities in patterns of metabolomic abundance, indicating that quenching can largely preserve cell metabolites. In contrast, storing cells at low temperature without quenching had significantly altered metabolite profiles (Group 3 vs Group 4). Although quenching can arrest cell metabolism, storage at as −80 °C for 48 h can still affect cell metabolites (Group 1 vs Group 3).

Trends observed from the heat maps are in generally good agreement with those obtained from PCA results. Our results indicate that the cells without quenching and after long-term storage (Group 4) had significantly altered metabolite profiles. Although storing quenched dried cells at −80 °C seems to be a reasonable choice, the storage time should be reduced to minimize the alternations of cell metabolites.

### Metabolites Changed Due to Omitted Quenching and during Storage

#### Positive Ion Mode Results

We investigated cell metabolites changed due to the omission of LN_2_ quenching by comparing the SCMS data obtained from cells in Group 1 vs Group 2 as well as Groups 3 vs Group 4. For the comparison of Group 1 and Group 2, abundances of 60 metabolites were significantly changed with 22 increased and 38 decreased metabolites (Figure S8A) (p-value < 0.05, FC > 1.5).^78^ Pathway analysis did not identify any significantly impacted pathways (i.e., FDR > 0.05) (Table S3). Studies were also conducted for HEK-293T cells prepared using the same procedures as those for HCT-116 cells in Groups 1 and 2. HEK-293T cells in these two groups also possess similar metabolomic profiles (Figure S9), and pathway analysis did not identify any significantly impacted pathway (Table S12). For the comparison of Group 3 and Group 4, abundances of 378 metabolites were significantly altered (324 increased and 53 decreased), as illustrated in the volcano plot (Figure S8B). We further conducted MS/MS analysis to identify those significantly altered ions at the single-cell level (Figure S4 and Table S1). The decreased metabolites include phospholipids (e.g., phosphatidylcholines (PC 43:11;O, PC 41:11;O, PC 32:0, PC O-35:8, and PC 30:0), lysophosphatidylcholine (LPC (34:0;O)), lysophosphatidic acid (LPA O-24:5), and glycerides (diglycerides (DG O-30:2). The increased metabolites include PCs (PC O-40:7, PC 36:2, PC O-34:2, PC 37:7, PC O-35:6), sphingomyelins (SM 45:1;O2), and cholesteryl esters (CE 18:3;O). Results from pathway analysis of significantly altered species, including both identified and tentatively labeled metabolites, resulted in multiple significantly changed pathways (Figure S10 and Table S4), including galactose metabolism, starch and sucrose metabolism, arachidonic acid metabolism, linoleic acid metabolism, biosynthesis of unsaturated fatty acids, and steroid biosynthesis pathway.

To evaluate the impact of the storage at −80 °C on cell metabolites, we performed comparisons between Group 1 and Group 3 as well as between Group 2 and Group 4. We discovered increased (58) and decreased (134) metabolites in the comparison of Group 1 vs Group 3 (Figure S8C). Pathway analysis based on tentatively labeled metabolites did not reveal any pathway significantly affected (Table S5). The comparison between Group 2 and Group 4 showed 144 increased and 100 decreased metabolites (Figure S8D). Pathway analysis based on tentatively labeled metabolites revealed that galactose metabolism and starch and sucrose metabolism were significantly impacted (Figure S11 and Table S6).

#### Negative Ion Mode Results

In alignment with the positive mode analysis, we performed similar comparisons using negative-ion mode data. To investigate cell metabolites changed due to the omission of LN_2_ quenching, we investigated the SCMS data obtained from cells in Group 1 vs Group 2 as well as Groups 3 vs Group 4. For comparison between Group 1 and Group 2, 145 metabolites were significantly changed with 20 increased and 125 decreased metabolites (Figure S12A). Pathway analysis revealed that galactose metabolism (Figure S13) significantly changed (Table S7). For comparison between Group 3 and Group 4, our results show that the abundances of 291 metabolites were significantly altered (Figure S12B), with 235 increased and 56 decreased metabolites. Using MS/MS analysis at the single-cell level, we identified multiple metabolites, including increased oleic acid and fatty acid FA 17:3;O4 as well as decreased triglycerides TG 51:14;O2 (Figure S5) (Table S1). Tentatively labeled species include decreased lipids (e.g., phosphatidylglycerols (PG 33:4, PG 43:4, PG 33:5, PG 32:4), DG 38:5, DG 35:4, and sphingomyelins (SM 36:5, SM 36:6) and increased organic acids (linoleic acid, succinic acid, and octadecenoic acid), lipid (MG 22:4), and other small molecules (alpha-d-glucose and creatine). Significantly altered metabolites in the comparison of Groups 3 and 4 indicate substantially affected pathways, suggesting that storing samples at −80 °C without quenching is insufficient to preserve the metabolomic integrity. Analysis of tentatively labeled metabolites revealed that 11 metabolic pathways were significantly affected due to storage without quenching (Table S8). These pathways include alanine, aspartate, and glutamate metabolism, d-amino acid metabolism, butanoate metabolism, linoleic acid metabolism, galactose metabolism, arginine and proline metabolism, valine, leucine, and isoleucine biosynthesis, valine, leucine, and isoleucine degradation, glycine, serine, and threonine metabolism, pantothenate and CoA biosynthesis, and caffeine metabolism (Figure S14).

To investigate the influence of low temperature storage on cell metabolites’ profiles, we performed the same comparison (Group 1 vs Group 3 and Group 2 vs Group 4). The comparison between Group 1 and Group 3 revealed 86 increased and 141 decreased metabolites (Figure S12C). Pathway analysis demonstrated that multiple pathways were significantly affected (Figure S15 and Table S9): arachidonic acid metabolism, arginine and proline metabolism, linoleic acid metabolism, d-amino acid metabolism, valine, leucine, and isoleucine biosynthesis, pantothenate and CoA biosynthesis, alanine, aspartate, and glutamate metabolism, and galactose metabolism. In the comparison between Group 2 and Group 4, 87 metabolites were increased, and 187 were decreased (Figure S12D). Three metabolic pathways were significantly impacted (Figure S16 and Table S10): arachidonic acid metabolism, valine, leucine, and isoleucine biosynthesis, and galactose metabolism. In this study, we integrated data from both positive and negative ion modes to enhance the coverage of detected metabolites and ensure a comprehensive metabolic pathway analysis. However, the integration did not reveal additional significantly altered metabolic pathways, indicating that the original analysis effectively captured the key metabolic changes under the experimental conditions.

Our results obtained from both positive and negative ion modes indicate LN_2_ quenching and freeze-drying are indispensable to preserve metabolites, but storage at low temperature (even at −80 °C) should be minimized to retain cell metabolites. Although rapid drying in a vacuum at room temperature can largely retain cell metabolites, cells need to be immediately analyzed after drying because storage at −80 °C can still change cell metabolites. Compared with freeze-drying, which forms small ice crystals with porous structures and large surface areas, drying at room temperatures is less effective to remove water molecules from cells.^79^ It is possible that residual water content in cells as well as the condensed water, which could be possibly formed during the defrosting process (e.g., during the transition from the −80 °C freezer to the desiccator and due to residual moisture in the desiccator), could result in partial rehydration of dried cells leading to reactions such as through reactivated enzymatic activities and hydrolysis reactions.

## Conclusion

In this study, live HCT-116 cells were washed with ammonium formate solution, quenched with LN_2_, freeze-dried in a vacuum, and stored in a −80 °C freezer. We then performed single-cell metabolomics studies using the Single-probe SCMS technique. Our results indicated that washing using ammonium formate led to enhancement in ion intensities attributed to the mitigated matrix effect. Remarkably, a diverse array of lipids, including PC, PS, PE, PA, PG, TG, DG, and MG, were identified from individual cells. We further studied the influence of LN_2_ quenching and storage at −80 °C on metabolites and metabolomic pathways. Notably, LN_2_ quenching and freeze-drying preserved cells’ metabolomic profiles. Storage of LN_2_ quenched cells at −80 °C for 48 h generally retained cell metabolites, enabling reliable SCMS experiments with extended time or low temperature shipped samples. However, the time delay between the LN_2_ quenching and SCMS experiments should be minimized. Although cells underwent rapid drying in vacuum at room temperature could largely retain metabolites, cells need to be immediately analyzed because storage (even at −80 °C for 48 h) could change metabolites’ compositions. These findings collectively contribute to the sample preparation techniques in single-cell metabolomics studies. The developed methods can be readily adopted by researchers using other ambient-based SCMS techniques for broad applications.

## Supplementary Material





## Data Availability

Raw data from SCMS experiments can be accessed in the MassIVE database (accession ID: MSV000096378). Python scripts for SCMS data alignment and power analysis are available on GitHub (https://github.com/dandandan001/SCMS-data-analysis).

## Notes

Results of random forest analysis of SCMS; pathway analysis; power analysis, volcano plots illustrating significantly changed species and tentatively labeled metabolites; significant peaks identified using MS/MS identification in single cells; previously published MS/MS spectra and in silico results; experimental SCMS setup of the Single-probe coupled to Thermo Orbitrap Exploris 240 mass spectrometer (PDF)
Summary of data analysis (XLSX)
