# Supplementary material for: Developing a Cell Quenching Method to Facilitate Single Cell Mass Spectrometry Metabolomics Studies
Source: JACS Au. 2025 May 6;5(5):2379–87. doi: 10.1021/jacsau.5c00327 (PMC12117403; doi:10.1021/jacsau.5c00327)
Supplement: Supplementary file 1 [file au5c00327_si_001.pdf]

Developing Cell Quenching Method to Facilitate Single Cell Mass Spectrometry Metabolomics Studies

Shakya Wije Munige[#a], Deepti Bhusal[#a], Zongkai Peng[a], Dan Chen[a], Zhibo Yang[*a,b]

[a] Department of Chemistry and Biochemistry, University of Oklahoma, Norman, Oklahoma, 73019, US

[b] Stephenson Cancer Center, University of Oklahoma Health Sciences Center, Oklahoma City, Oklahoma, 73104, US

[#]These authors contributed equally

[*] Corresponding author

Email: Zhibo.Yang@ou.edu

Table of Contents

Supporting Tables	S3
Supporting Figures	S18
Post hoc power analysis	S38
References	S39

Supporting Tables

Table S1. MS/MS identified metabolites significantly changed in positive and negative ion modes. *

m/z	Molecular Formula	Adduct	Compound	NCE HCD
882.5668**	$C_{51}H_{80}NO_9P$	[M+H]+	PC 43:11;O	10
879.7378	$C_{50}H_{101}N_2O_6P$	[M+Na]+	SM 45:1;O2	30
856.5645	$C_{48}H_{84}NO_7P$	[M+K]+	PC O-40:7	10
854.5352**	$C_{49}H_{76}NO_9P$	[M+H]+	PC 41:11;O	10
812.5152	$C_{45}H_{76}NO_8P$	[M+Na]+	PC 37:7	25
808.5793	$C_{44}H_{84}NO_8P$	[M+Na]+	PC 36:2	20
772.6113**	$C_{42}H_{88}NO_7P$	[M+Na]+	LPC O-34:0	25
772.5226	$C_{43}H_{76}NO_7P$	[M+Na]+	PC O-35:6	20
768.491**	$C_{43}H_{72}NO_7P$	[M+Na]+	PC O-35:8	20
744.5875	$C_{42}H_{82}NO_7P$	[M+H]+	PC 34:2	18
734.5674**	$C_{40}H_{80}NO_8P$	[M+H]+	PC 32:0	20
706.5353	$C_{38}H_{76}NO_8P$	[M+H]+	PC 30:0	20
685.5505	$C_{45}H_{74}O_3$	[M+Na]+	CE 18:3;O	20
537.2759**	$C_{27}H_{47}O_6P$	[M+K]+	LPA O-24:5	25
523.4705	$C_{33}H_{62}O_4$	[M+Na]+	DG 30:2	15
281.2482	$C_{18}H_{34}O_2$	[M-H]-	Oleic acid	35
327.18	$C_{17}H_{28}O_6$	[M-H]-	FA 17:3;O4	20
425.2712**	$C_{54}H_{76}O_8$	[M-2H]2-	TG 51:14;O2	25

*Features in Figures S2 and S7 were identified using MS/MS analysis of positive (red font) and negative (blue font) ions at the single-cell level.
**Upregulated metabolites.

Table S2. Random forest analysis of SCMS results obtained from the positive ion mode.

	Group 1	Group 2	Group 3	Group 4	Classification Error
Group 1	25	3	4	2	0.27
Group 2	7	18	5	2	0.44
Group 3	7	3	22	2	0.33
Group 4	1	1	3	27	0.16

Table S3. Pathway analysis of Group 1 vs. Group 2 SCMS results in the positive ion mode. *

Pathways	Total	Expected	Hits	Raw p	-log10(p)	Holm adjust	FDR	Impact
Steroid hormone biosynthesis	87	0.55238	4	0.0014136	2.8497	0.11309	0.11309	0.06273
Ubiquinone and other terpenoid-quinone biosynthesis	18	0.11429	2	0.0052607	2.279	0.41559	0.21043	0.38462
Sphingolipid metabolism	32	0.20317	2	0.016262	1.7888	1	0.43364	0.35835
Linoleic acid metabolism	5	0.031746	1	0.031385	1.5033	1	0.6277	0
Glycerophospholipid metabolism	36	0.22857	1	0.20697	0.68409	1	1	0.01896

*HCT-116 cells were washed by ammonium formate solution and dried in a vacuum. No metabolomic pathway was affected significantly (FDR < 0.05) by rapid drying at room temperature (RT) without storage.

Table S4. Pathway analysis of Group 3 vs. Group 4 SCMS results in the positive ion mode.*

Pathways	Total	Expected	Hits	Raw p	-log10(p)	Holm adjust	FDR	Impact
**Galactose metabolism	27	1.3371	11	1.4907E-08	7.8266	1.1925E-06	1.1925E-06	0.70254
Starch and sucrose metabolism	18	0.89143	7	1.1453E-05	4.9411	0.00090478	0.00045812	0.54856
**Arachidonic acid metabolism	44	2.179	8	0.0010475	2.9798	0.081705	0.021767	0.37522
**Linoleic acid metabolism	5	0.24762	3	0.0010883	2.9632	0.083803	0.021767	1
**Biosynthesis of unsaturated fatty acids	36	1.7829	7	0.0014544	2.8373	0.11053	0.02327	0
**Steroid biosynthesis	41	2.0305	7	0.003204	2.4943	0.2403	0.04272	0.16089
Drug metabolism - cytochrome P450	55	2.7238	6	0.050712	1.2949	1	0.57957	0.1087
Steroid hormone biosynthesis	87	4.3086	8	0.061136	1.2137	1	0.58071	0.1903

Ether lipid metabolism	20	0.99048	3	0.072589	1.1391	1	0.58071	0
Fructose and mannose metabolism	20	0.99048	3	0.072589	1.1391	1	0.58071	0.09765
Caffeine metabolism	10	0.49524	2	0.084194	1.0747	1	0.60722	0.69231
Neomycin, kanamycin and gentamicin biosynthesis	2	0.099048	1	0.096625	1.0149	1	0.60722	0
Glycerophospholipid metabolism	36	1.7829	4	0.098674	1.0058	1	0.60722	0.09383
alpha-Linolenic acid metabolism	13	0.64381	2	0.13274	0.877	1	0.75852	0.33333
Ubiquinone and other terpenoid-quinone biosynthesis	18	0.89143	2	0.22293	0.65183	1	1	0.23077
Purine metabolism	70	3.4667	5	0.26305	0.57995	1	1	0.13487
Nitrogen metabolism	6	0.29714	1	0.26306	0.57994	1	1	0
Taurine and hypotaurine metabolism	8	0.39619	1	0.33453	0.47556	1	1	0
Amino sugar and nucleotide sugar metabolism	42	2.08	3	0.34557	0.46147	1	1	0
Ascorbate and aldarate metabolism	9	0.44571	1	0.36766	0.43456	1	1	0
Sphingolipid metabolism	32	1.5848	2	0.47679	0.32167	1	1	0.35835
Arginine biosynthesis	14	0.69333	1	0.51038	0.29211	1	1	0
D-Amino acid metabolism	15	0.74286	1	0.53484	0.27177	1	1	0
Retinol metabolism	17	0.8419	1	0.58021	0.23642	1	1	0.15464
Terpenoid backbone biosynthesis	18	0.89143	1	0.60123	0.22096	1	1	0.18254
Pantothenate and CoA biosynthesis	20	0.99048	1	0.64019	0.19369	1	1	0.0068
Glycolysis / Gluconeogenesis	26	1.2876	1	0.73591	0.13317	1	1	0.00944
Folate biosynthesis	27	1.3371	1	0.74921	0.1254	1	1	0
Alanine, aspartate and glutamate metabolism	28	1.3867	1	0.76185	0.11813	1	1	0.11378
Glutathione metabolism	28	1.3867	1	0.76185	0.11813	1	1	0.25596
Inositol phosphate metabolism	30	1.4857	1	0.78526	0.10498	1	1	0.12939
Glyoxylate and dicarboxylate metabolism	32	1.5848	1	0.8064	0.093447	1	1	0
Fatty acid elongation	39	1.9314	1	0.86545	0.062758	1	1	0
Fatty acid degradation	39	1.9314	1	0.86545	0.062758	1	1	0
Pyrimidine metabolism	39	1.9314	1	0.86545	0.062758	1	1	0
Tyrosine metabolism	42	2.08	1	0.88494	0.053087	1	1	0.00635
Primary bile acid biosynthesis	46	2.2781	1	0.90665	0.042562	1	1	0.05573
Fatty acid biosynthesis	47	2.3276	1	0.91141	0.040286	1	1	0.01473

* HCT-116 cells were washed by ammonium formate solution, dried, and stored at -80℃ (48h).

**Metabolomic pathways significantly altered (FDR < 0.05) due to omitted LN_2 quenching.

Table S5. Pathway analysis of Group 1 vs. Group 3 SCMS results in the positive ion mode. *

Pathways	Total	Expected	Hits	Raw p	-log10(p)	Holm adjust	FDR	Impact
Metabolism of xenobiotics by cytochrome P450	68	0.47492	4	0.0008349	3.0784	0.066792	0.066792	0.09183
Biosynthesis of unsaturated fatty acids	36	0.25143	2	0.024549	1.61	1	0.98195	0
Purine metabolism	70	0.48889	2	0.082653	1.0827	1	1	0.00565
alpha-Linolenic acid metabolism	13	0.090794	1	0.087404	1.0585	1	1	0.33333
Glycerophospholipid metabolism	36	0.25143	1	0.22521	0.64741	1	1	0.01896
Steroid biosynthesis	41	0.28635	1	0.25254	0.59766	1	1	0
Primary bile acid biosynthesis	46	0.32127	1	0.279	0.5544	1	1	0.00016

* HCT-116 cells were washed by ammonium formate solution, LN_2 quenched, and dried. No metabolomic pathway was affected significantly (FDR < 0.05) by storage at -80°C (48h).

Table S6. Pathway analysis of Group 2 vs. Group 4 SCMS results in the positive ion mode.*

Pathways	Total	Expected	Hits	Raw p	-log10(p)	Holm adjust	FDR	Impact
**Galactose metabolism	27	0.75429	11	2.042E-11	10.69	1.6336E-09	1.6336E-09	0.70254
**Starch and sucrose metabolism	18	0.50286	7	2.0591E-07	6.6863	1.6267E-05	8.2362E-06	0.54856
Arachidonic acid metabolism	44	1.2292	5	0.006511	2.1864	0.50786	0.17363	0.02913
Fructose and mannose metabolism	20	0.55873	3	0.016643	1.7788	1	0.33286	0.09765
Caffeine metabolism	10	0.27937	2	0.029775	1.5262	1	0.47639	0.69231

Neomycin, kanamycin and gentamicin biosynthesis	2	0.055873	1	0.05511	1.2588	1	0.7348	0
Amino sugar and nucleotide sugar metabolism	42	1.1733	3	0.10977	0.9595	1	1	0
Nitrogen metabolism	6	0.16762	1	0.15657	0.80529	1	1	0
Drug metabolism - cytochrome P450	55	1.5365	3	0.19638	0.70689	1	1	0.02174
Taurine and hypotaurine metabolism	8	0.22349	1	0.20323	0.69202	1	1	0
Ascorbate and aldarate metabolism	9	0.25143	1	0.2256	0.64666	1	1	0
Glycerophospholipid metabolism	36	1.0057	2	0.26624	0.57473	1	1	0.05751
Pyrimidine metabolism	39	1.0895	2	0.29785	0.526	1	1	0.03985
alpha-Linolenic acid metabolism	13	0.36317	1	0.30911	0.50989	1	1	0
Purine metabolism	70	1.9556	3	0.3106	0.50779	1	1	0.00565
Steroid biosynthesis	41	1.1454	2	0.31885	0.49642	1	1	0.00346
Arginine biosynthesis	14	0.39111	1	0.32857	0.48337	1	1	0
D-Amino acid metabolism	15	0.41905	1	0.3475	0.45905	1	1	0
Primary bile acid biosynthesis	46	1.2851	2	0.37067	0.43101	1	1	0.11029
Ubiquinone and other terpenoid-quinone biosynthesis	18	0.50286	1	0.4012	0.39664	1	1	0.23077
Ether lipid metabolism	20	0.55873	1	0.43458	0.36193	1	1	0
Pantothenate and CoA biosynthesis	20	0.55873	1	0.43458	0.36193	1	1	0.0068
Glycolysis / Gluconeogenesis	26	0.72635	1	0.52417	0.28053	1	1	0.00944
Folate biosynthesis	27	0.75429	1	0.53768	0.26948	1	1	0
Alanine, aspartate and glutamate metabolism	28	0.78222	1	0.55082	0.25899	1	1	0.11378
Glutathione metabolism	28	0.78222	1	0.55082	0.25899	1	1	0.25596
Inositol phosphate metabolism	30	0.8381	1	0.57602	0.23956	1	1	0.12939
Glyoxylate and dicarboxylate metabolism	32	0.89397	1	0.59983	0.22197	1	1	0
Sphingolipid metabolism	32	0.89397	1	0.59983	0.22197	1	1	0.21576
Biosynthesis of unsaturated fatty acids	36	1.0057	1	0.6436	0.19138	1	1	0

*HCT-116 cells were washed by ammonium formate solution and dried.

**Metabolomic pathways significantly altered (FDR < 0.05) due to omitted LN_2 quenching.

Table S7. Pathway analysis of Group 1 vs. Group 2 SCMS results in the negative ion mode.*

Pathways	Total	Expected	Hits	Raw p	-log10(p)	Holm adjust	FDR	Impact
**Galactose metabolism	27	0.44571	6	2.5748e-06	5.5893	0.000206	0.000206	0.42037
Fructose and mannose metabolism	20	0.33016	3	0.003782	2.4223	0.29877	0.10085	0.09765
Citrate cycle (TCA cycle)	20	0.33016	3	0.003782	2.4223	0.29877	0.10085	0.16809
Alanine, aspartate and glutamate metabolism	28	0.46222	3	0.009955	2.002	0.76651	0.174	0
Caffeine metabolism	10	0.16508	2	0.010875	1.9636	0.82649	0.174	0.69231
Histidine metabolism	16	0.26413	2	0.027289	1.564	1	0.30309	0.04918
Amino sugar and nucleotide sugar metabolism	42	0.69333	3	0.029932	1.5239	1	0.30309	0
Neomycin, kanamycin and gentamicin biosynthesis	2	0.033016	1	0.032754	1.4847	1	0.30309	0
Starch and sucrose metabolism	18	0.29714	2	0.034098	1.4673	1	0.30309	0.42527
Glyoxylate and dicarboxylate metabolism	32	0.52825	2	0.096066	1.0174	1	0.7167	0.03175
Metabolism of xenobiotics by cytochrome P450	68	1.1225	3	0.098546	1.0064	1	0.7167	0
Pyrimidine metabolism	39	0.64381	2	0.13389	0.87325	1	0.79692	0.08894
Drug metabolism - other enzymes	39	0.64381	2	0.13389	0.87325	1	0.79692	0.13043
Ascorbate and aldarate metabolism	9	0.14857	1	0.13946	0.85555	1	0.79692	0
Butanoate metabolism	15	0.24762	1	0.22183	0.65398	1	1	0
Nicotinate and nicotinamide metabolism	15	0.24762	1	0.22183	0.65398	1	1	0
Pantothenate and CoA biosynthesis	20	0.33016	1	0.28463	0.54572	1	1	0
beta-Alanine metabolism	21	0.34667	1	0.29659	0.52784	1	1	0
Propanoate metabolism	22	0.36317	1	0.30836	0.51094	1	1	0
Glycolysis / Gluconeogenesis	26	0.42921	1	0.35357	0.45152	1	1	0.00944
Inositol phosphate metabolism	30	0.49524	1	0.39593	0.40238	1	1	0.12939
Valine, leucine and isoleucine degradation	40	0.66032	1	0.49048	0.30938	1	1	0.02264

Arachidonic acid metabolism	44	0.72635	1	0.52417	0.28053	1	1	0
Purine metabolism	70	1.1556	1	0.6963	0.1572	1	1	0.01146
Steroid hormone biosynthesis	87	1.4362	1	0.77453	0.11096	1	1	0

*HCT-116 cells were washed by ammonium formate solution and dried.

**Metabolomic pathways significantly altered (FDR < 0.05) due to omitted LN_2 quenching.

Table S8. Pathway analysis of Group 3 vs. Group 4 SCMS results in the negative ion mode.*

Pathways	Total	Expected	Hits	Raw p	-log10(p)	Holm adjust	FDR	Impact
**Alanine, aspartate and glutamate metabolism	28	1.9733	11	1.0159E-06	5.9931	8.1276E-05	8.085E-05	0.75562
**D-Amino acid metabolism	15	1.0571	8	2.0212E-06	5.6944	0.00015968	8.085E-05	1
**Butanoate metabolism	15	1.0571	7	2.8803E-05	4.5406	0.0022466	0.00076807	0.17461
**Linoleic acid metabolism	5	0.35238	4	0.00011085	3.9553	0.0085355	0.002217	1
**Galactose metabolism	27	1.9029	8	0.00033944	3.4692	0.025798	0.0054311	0.42037
**Arginine and proline metabolism	36	2.5371	9	0.00058114	3.2357	0.043586	0.0077486	0.15697
**Valine, leucine and isoleucine biosynthesis	8	0.56381	4	0.0013127	2.8818	0.097137	0.013182	0
**Valine, leucine and isoleucine degradation	40	2.819	9	0.0013271	2.8771	0.097137	0.013182	0.09069
**Glycine, serine and threonine metabolism	33	2.3257	8	0.001483	2.8289	0.10677	0.013182	0.3839
**Pantothenate and CoA biosynthesis	20	1.4095	6	0.0018358	2.7362	0.13034	0.014686	0.12245
**Caffeine metabolism	10	0.70476	4	0.0035241	2.453	0.24668	0.02563	0.69231
beta-Alanine metabolism	21	1.48	5	0.012985	1.8866	0.89597	0.086567	0.5597
Propanoate metabolism	22	1.5505	5	0.015879	1.7992	1	0.095215	0.04103

Pyrimidine metabolism	39	2.7486	7	0.016663	1.7783	1	0.095215	0.12673
Histidine metabolism	16	1.1276	4	0.021952	1.6585	1	0.11708	0.04918
Phenylalanine, tyrosine and tryptophan biosynthesis	4	0.2819	2	0.026892	1.5704	1	0.13446	1
Fructose and mannose metabolism	20	1.4095	4	0.047012	1.3278	1	0.22124	0.13078
Nitrogen metabolism	6	0.42286	2	0.061245	1.2129	1	0.2722	0
Arginine biosynthesis	14	0.98667	3	0.070106	1.1542	1	0.29518	0.11675
Nicotinate and nicotinamide metabolism	15	1.0571	3	0.083285	1.0794	1	0.33314	0
Biosynthesis of unsaturated fatty acids	36	2.5371	5	0.10401	0.98291	1	0.37914	0
Phenylalanine metabolism	8	0.56381	2	0.10426	0.98187	1	0.37914	0.35714
Drug metabolism - other enzymes	39	2.7486	5	0.13474	0.87051	1	0.45342	0.20651
Neomycin, kanamycin and gentamicin biosynthesis	2	0.14095	1	0.13603	0.86637	1	0.45342	0
Glyoxylate and dicarboxylate metabolism	32	2.2552	4	0.18424	0.73462	1	0.58957	0.04233
Metabolism of xenobiotics by cytochrome P450	68	4.7924	6	0.34508	0.46208	1	1	0
Starch and sucrose metabolism	18	1.2686	2	0.36586	0.43669	1	1	0.42527
Arachidonic acid metabolism	44	3.101	4	0.37621	0.42458	1	1	0.34609
Cysteine and methionine metabolism	33	2.3257	3	0.41461	0.38237	1	1	0.12346
Citrate cycle (TCA cycle)	20	1.4095	2	0.41735	0.3795	1	1	0.07685
Taurine and hypotaurine metabolism	8	0.56381	1	0.44346	0.35315	1	1	0.42857
Ascorbate and aldarate metabolism	9	0.63429	1	0.48288	0.31616	1	1	0
Purine metabolism	70	4.9333	5	0.55744	0.2538	1	1	0.09122

Folate biosynthesis	27	1.9029	2	0.57855	0.23766	1	1	0.09924
Amino sugar and nucleotide sugar metabolism	42	2.96	3	0.5786	0.23762	1	1	0
Tyrosine metabolism	42	2.96	3	0.5786	0.23762	1	1	0.13972
Glutathione metabolism	28	1.9733	2	0.59879	0.22273	1	1	0.04258
Primary bile acid biosynthesis	46	3.2419	3	0.64179	0.1926	1	1	0.00774
Porphyrin metabolism	31	2.1848	2	0.65514	0.18367	1	1	0.02795
Retinol metabolism	17	1.1981	1	0.71321	0.14678	1	1	0.15464
Ubiquinone and other terpenoid-quinone biosynthesis	18	1.2686	1	0.73364	0.13452	1	1	0
Terpenoid backbone biosynthesis	18	1.2686	1	0.73364	0.13452	1	1	0.11429
Pentose and glucuronate interconversions	19	1.339	1	0.75263	0.12342	1	1	0
Selenocompound metabolism	20	1.4095	1	0.77028	0.11335	1	1	0
Ether lipid metabolism	20	1.4095	1	0.77028	0.11335	1	1	0
Tryptophan metabolism	41	2.8895	2	0.7986	0.097669	1	1	0.03184
Pyruvate metabolism	23	1.621	1	0.81607	0.088272	1	1	0.0283
Glycolysis / Gluconeogenesis	26	1.8324	1	0.8528	0.069152	1	1	0.00944
Lipoic acid metabolism	28	1.9733	1	0.87315	0.058912	1	1	0.06136
Inositol phosphate metabolism	30	2.1143	1	0.8907	0.050266	1	1	0.12939
Lysine degradation	30	2.1143	1	0.8907	0.050266	1	1	0.13429
Sphingolipid metabolism	32	2.2552	1	0.90585	0.042944	1	1	0
Drug metabolism - cytochrome P450	55	3.8762	2	0.91103	0.040466	1	1	0.01449
Glycerophospholipid metabolism	36	2.5371	1	0.93018	0.031434	1	1	0.04289
Steroid biosynthesis	41	2.8895	1	0.952	0.021362	1	1	0
Fatty acid biosynthesis	47	3.3124	1	0.96944	0.01348	1	1	0

*HCT-116 cells were washed by ammonium formate solution, dried, and stored at -80℃ (48h).

**Metabolomic pathways significantly altered (FDR < 0.05) due to omitted LN_2 quenching.

Table S9. Pathway analysis of Group 1 vs. Group 3 SCMS results in the negative ion mode.*

Pathways	Total	Expected	Hits	Raw p	-log10(p)	Holm adjust	FDR	Impact
**Arachidonic acid metabolism	44	2.5422	15	4.9516E-09	8.3053	3.9613E-07	3.9613E-07	0.27518
**Arginine and proline metabolism	36	2.08	10	1.8508E-05	4.7326	0.0014621	0.00074032	0.18023
**Linoleic acid metabolism	5	0.28889	4	5.0001E-05	4.301	0.0039001	0.0013334	1
**D-Amino acid metabolism	15	0.86667	6	0.00010365	3.9844	0.0079809	0.002073	1
**Valine, leucine and isoleucine biosynthesis	8	0.46222	4	0.00061128	3.2138	0.046457	0.0084496	0
**Pantothenate and CoA biosynthesis	20	1.1556	6	0.00063372	3.1981	0.047529	0.0084496	0.12245
**Alanine, aspartate and glutamate metabolism	28	1.6178	7	0.00074502	3.1278	0.055131	0.0085145	0.48398
**Galactose metabolism	27	1.56	6	0.0034808	2.4583	0.2541	0.034808	0.42037
Glycine, serine and threonine metabolism	33	1.9067	6	0.009832	2.0074	0.7079	0.087395	0.2208
Biosynthesis of unsaturated fatty acids	36	2.08	6	0.015031	1.823	1	0.12025	0
Phenylalanine, tyrosine and tryptophan biosynthesis	4	0.23111	2	0.018358	1.7362	1	0.13351	1
Pyrimidine metabolism	39	2.2533	6	0.021895	1.6597	1	0.14597	0.0904
beta-Alanine metabolism	21	1.2133	4	0.029267	1.5336	1	0.18011	0.5597
Nitrogen metabolism	6	0.34667	2	0.042536	1.3712	1	0.24306	0
Butanoate metabolism	15	0.86667	3	0.051207	1.2907	1	0.2731	0.03175
Phenylalanine metabolism	8	0.46222	2	0.07364	1.1329	1	0.36001	0.35714
Valine, leucine and isoleucine degradation	40	2.3111	5	0.076503	1.1163	1	0.36001	0
Fructose and mannose metabolism	20	1.1556	3	0.10438	0.98139	1	0.42763	0.09765

Glyoxylate and dicarboxylate metabolism	32	1.8489	4	0.10885	0.96319	1	0.42763	0.03175
Caffeine metabolism	10	0.57778	2	0.10984	0.95922	1	0.42763	0.69231
Neomycin, kanamycin and gentamicin biosynthesis	2	0.11556	1	0.11225	0.94981	1	0.42763	0
Arginine biosynthesis	14	0.80889	2	0.19178	0.71719	1	0.69739	0.11675
Drug metabolism - cytochrome P450	55	3.1778	5	0.20813	0.68166	1	0.72394	0.03623
Starch and sucrose metabolism	18	1.04	2	0.27925	0.55401	1	0.93083	0.42527
Citrate cycle (TCA cycle)	20	1.1556	2	0.3231	0.49066	1	1	0.13536
Taurine and hypotaurine metabolism	8	0.46222	1	0.37949	0.4208	1	1	0.42857
Ascorbate and aldarate metabolism	9	0.52	1	0.41552	0.38141	1	1	0
Amino sugar and nucleotide sugar metabolism	42	2.4267	3	0.44186	0.35472	1	1	0
Folate biosynthesis	27	1.56	2	0.46898	0.32884	1	1	0.09924
Glutathione metabolism	28	1.6178	2	0.48836	0.31126	1	1	0.02675
Primary bile acid biosynthesis	46	2.6578	3	0.5031	0.29835	1	1	0.00774
alpha-Linolenic acid metabolism	13	0.75111	1	0.5401	0.26753	1	1	0.33333
Porphyrin metabolism	31	1.7911	2	0.5438	0.26456	1	1	0.02795
Nicotinate and nicotinamide metabolism	15	0.86667	1	0.59214	0.22758	1	1	0
Histidine metabolism	16	0.92444	1	0.61593	0.21047	1	1	0
Ubiquinone and other terpenoid-quinone biosynthesis	18	1.04	1	0.65947	0.1808	1	1	0
Pentose and glucuronate interconversions	19	1.0978	1	0.67937	0.16789	1	1	0
Selenocompound metabolism	20	1.1556	1	0.69813	0.15607	1	1	0

Propanoate metabolism	22	1.2711	1	0.73243	0.13523	1	1	0
Purine metabolism	70	4.0444	3	0.78354	0.10594	1	1	0.01146
Glycolysis / Gluconeogenesis	26	1.5022	1	0.7899	0.10243	1	1	0.00944
Lysine degradation	30	1.7333	1	0.83513	0.078247	1	1	0.11247
Inositol phosphate metabolism	30	1.7333	1	0.83513	0.078247	1	1	0.12939
Cysteine and methionine metabolism	33	1.9067	1	0.86259	0.064193	1	1	0.04179
Fatty acid elongation	39	2.2533	1	0.90467	0.043511	1	1	0
Fatty acid degradation	39	2.2533	1	0.90467	0.043511	1	1	0
Drug metabolism - other enzymes	39	2.2533	1	0.90467	0.043511	1	1	0
Steroid biosynthesis	41	2.3689	1	0.91563	0.038279	1	1	0
Tyrosine metabolism	42	2.4267	1	0.92064	0.035911	1	1	0.13972
Fatty acid biosynthesis	47	2.7156	1	0.94158	0.026142	1	1	0.01473
Metabolism of xenobiotics by cytochrome P450	68	3.9289	1	0.98405	0.0069824	1	1	0.05612

*HCT116 cells were washed by ammonium formate solution, quenched by LN_2, and freeze-dried in a vacuum.

**Metabolomic pathways significantly altered (FDR < 0.05) due to storage at -80°C (48h).

Table S10. Pathway analysis of Group 2 vs. Group 4 SCMS results in the negative ion mode.*

Pathway	Total	Expected	Hits	Raw p	-log10(p)	Holm adjust	FDR	Impact
**Arachidonic acid metabolism	44	2.6819	16	1.0783E-09	8.9673	8.6263E-08	8.6263E-08	0.27518
**Valine, leucine and isoleucine biosynthesis	8	0.48762	4	0.00075176	3.1239	0.059389	0.021719	0
**Galactose metabolism	27	1.6457	7	0.00081447	3.0891	0.063529	0.021719	0.45614
Biosynthesis of unsaturated fatty acids	36	2.1943		0.0048618	2.3132	0.37436	0.097236	0
Arginine and proline metabolism	36	2.1943	6	0.019229	1.716	1	0.27138	0.1093

Phenylalanine, tyrosine and tryptophan biosynthesis	4	0.24381	2	0.020353	1.6914	1	0.27138	1
Alanine, aspartate and glutamate metabolism	28	1.7067	5	0.024295	1.6145	1	0.27766	0.08654
Pantothenate and CoA biosynthesis	20	1.219	4	0.029529	1.5298	1	0.29529	0.05442
Glycine, serine and threonine metabolism	33	2.0114	5	0.04614	1.3359	1	0.41013	0.14654
D-Amino acid metabolism	15	0.91429	3	0.058508	1.2328	1	0.46807	0
Phenylalanine metabolism	8	0.48762	2	0.080952	1.0918	1	0.56591	0.35714
Drug metabolism - cytochrome P450	55	3.3524	6	0.11329	0.94583	1	0.56591	0.03623
Metabolism of xenobiotics by cytochrome P450	68	4.1448	7	0.115	0.93929	1	0.56591	0.05612
Fructose and mannose metabolism	20	1.219	3	0.11792	0.92842	1	0.56591	0.09765
Citrate cycle (TCA cycle)	20	1.219	3	0.11792	0.92842	1	0.56591	0.16809
Neomycin, kanamycin and gentamicin biosynthesis	2	0.1219	1	0.11823	0.92729	1	0.56591	0
Caffeine metabolism	10	0.60952	2	0.12026	0.91989	1	0.56591	0.69231
Pyrimidine metabolism	39	2.3771	4	0.21046	0.67682	1	0.93539	0.09684
Valine, leucine and isoleucine degradation	40	2.4381	4	0.22366	0.65041	1	0.94173	0.02264
Histidine metabolism	16	0.97524	2	0.25455	0.59423	1	1	0.04918
Linoleic acid metabolism	5	0.30476	1	0.27011	0.56846	1	1	1
Starch and sucrose metabolism	18	1.0971	2	0.30103	0.5214	1	1	0.42527
Fatty acid biosynthesis	47	2.8648	4	0.32075	0.49383	1	1	0.01473
beta-Alanine metabolism	21	1.28	2	0.3697	0.43215	1	1	0.39925
Propanoate metabolism	22	1.341	2	0.39205	0.40665	1	1	0
Taurine and hypotaurine metabolism	8	0.48762	1	0.39605	0.40225	1	1	0.42857

Drug metabolism - other enzymes	39	2.3771	3	0.42846	0.36809	1	1	0.16847
Ascorbate and aldarate metabolism	9	0.54857	1	0.43305	0.36346	1	1	0
Amino sugar and nucleotide sugar metabolism	42	2.56	3	0.4777	0.32084	1	1	0
Folate biosynthesis	27	1.6457	2	0.49782	0.30293	1	1	0.09924
Primary bile acid biosynthesis	46	2.8038	3	0.54019	0.26745	1	1	0.00774
Lysine degradation	30	1.8286	2	0.55551	0.25531	1	1	0.24676
alpha-Linolenic acid metabolism	13	0.79238	1	0.55992	0.25188	1	1	0.33333
Glyoxylate and dicarboxylate metabolism	32	1.9505	2	0.59133	0.22817	1	1	0.03175
Butanoate metabolism	15	0.91429	1	0.61237	0.21299	1	1	0
Nicotinate and nicotinamide metabolism	15	0.91429	1	0.61237	0.21299	1	1	0
Ubiquinone and other terpenoid-quinone biosynthesis	18	1.0971	1	0.67966	0.16771	1	1	0
Terpenoid backbone biosynthesis	18	1.0971	1	0.67966	0.16771	1	1	0.11429
Selenocompound metabolism	20	1.219	1	0.71795	0.1439	1	1	0
Ether lipid metabolism	20	1.219	1	0.71795	0.1439	1	1	0
Glycolysis / Gluconeogenesis	26	1.5848	1	0.80769	0.092754	1	1	0.00944
Lipoic acid metabolism	28	1.7067	1	0.8308	0.080505	1	1	0.06136
Inositol phosphate metabolism	30	1.8286	1	0.85115	0.069993	1	1	0.12939
Porphyrin metabolism	31	1.8895	1	0.8604	0.065299	1	1	0.02795
Glycerophospholipid metabolism	36	2.1943	1	0.89877	0.04635	1	1	0.04289
Fatty acid elongation	39	2.3771	1	0.91657	0.037835	1	1	0
Fatty acid degradation	39	2.3771	1	0.91657	0.037835	1	1	0
Steroid biosynthesis	41	2.499	1	0.92668	0.033072	1	1	0
Tyrosine metabolism	42	2.56	1	0.93126	0.030927	1	1	0.13972
Purine metabolism	70	4.2667	2	0.93626	0.028604	1	1	0.01146
Steroid hormone biosynthesis	87	5.3029	1	0.99642	0.0015588	1	1	0

*HCT-116 cells were washed by ammonium formate solution and freeze-dried in a vacuum.
**Metabolomic pathways significantly altered (FDR < 0.05) due to storage at -80°C (48h) without LN_2 quenching.

Table S11. Random forest analysis of SCMS results obtained from the negative ion mode.

	Group 1	Group 2	Group 3	Group 4	Classification Error
Group 1	24	7	1	0	0.25
Group 2	7	22	2	0	0.29
Group 3	1	1	25	3	0.17
Group 4	1	0	0	33	0.03

Table S12. Pathway analysis of SCMS results (positive ion mode) obtained from HEK 293T cells in Group 1 vs. Group 2.*

Pathways	Total	Expected	Hits	Raw p	-log10(p)	Holm adjust	FDR	Impact
Sphingolipid metabolism	32	0.060302	2	0.0011602	2.9355	0.092814	0.092814	0
Glycerophospholipid metabolism	36	0.067839	1	0.066357	1.1781	1	1	0.01739

*No metabolomic pathway was affected significantly (FDR > 0.05) by rapid drying at room temperature (RT) without storage. HEK 293T cells in Group 1 and Group 2 were prepared using the same procedures as HCT-116 cells in Group 1 and Group 2.

Supporting Figures

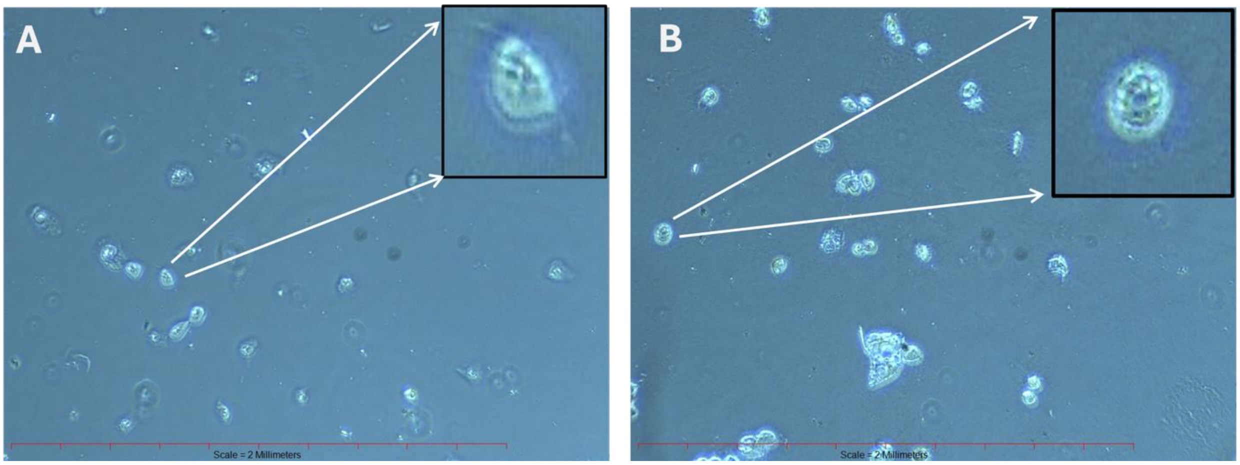

Figure S1. Microscopic view (4x) of HCT116 (A) before and (B) after LN_2 quenching, freeze drying, and storage in -80°C freezer.

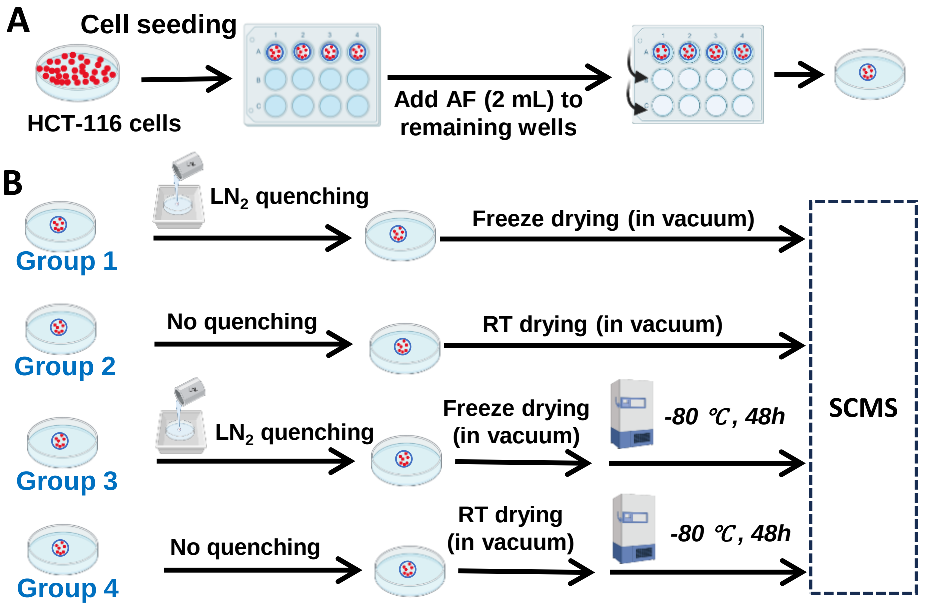

Figure S2. Overall workflow of SCMS studies of the impact of LN_2 quenching and -80 ℃ storage (48 h) on metabolites' profiles in single cells. (A) Cell seeding and washing by AF (ammonium formate) solution. (B) Four groups of cells were used in experiments. Group 1 – Cells were washed, quenched, and freeze dried (no storage); Group 2 – Cells were washed and dried at room temperature (RT)) (no quenching and storage); Group 3 – Cells were quenched, freeze dried, and stored; Group 4 – Cells were dried at RT and stored (no quenching).

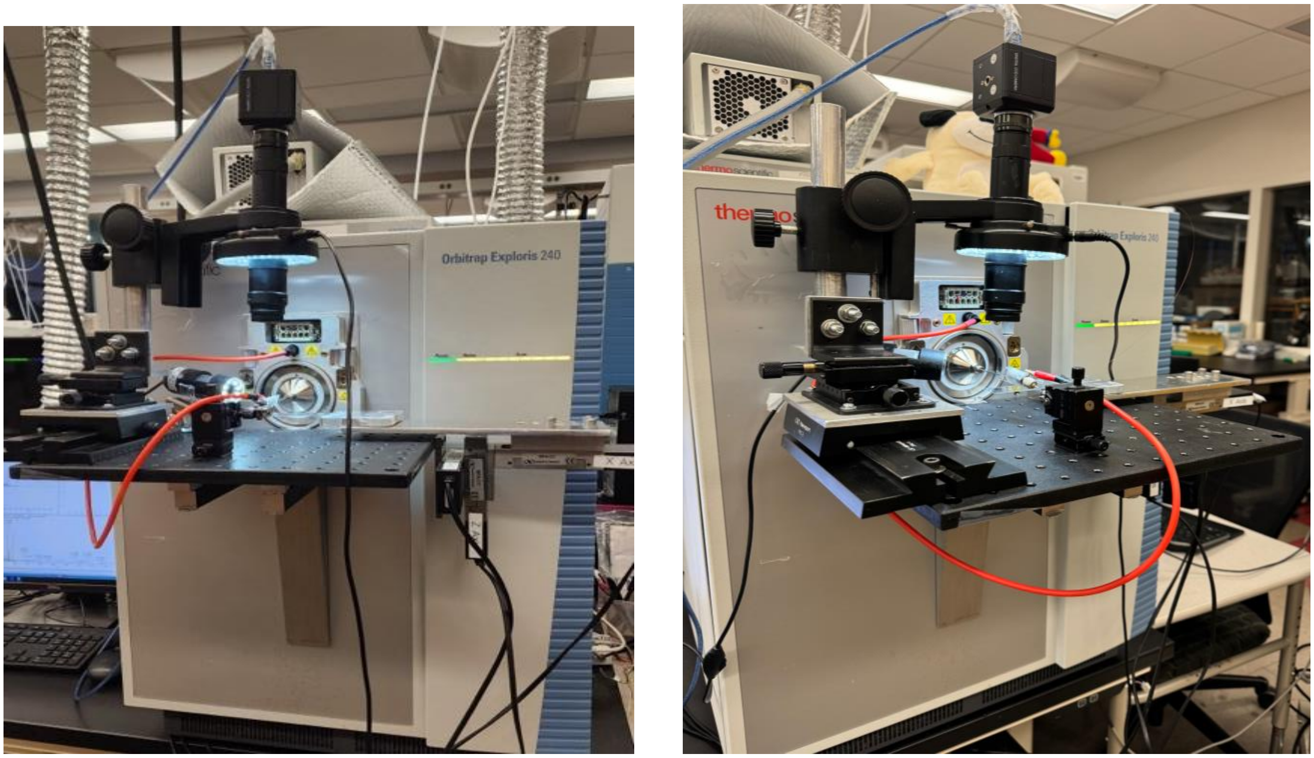

Figure S3. The Single-probe device coupled to a Thermo Orbitrap Exploris 240 mass spectrometer for SCMS experiments.

A

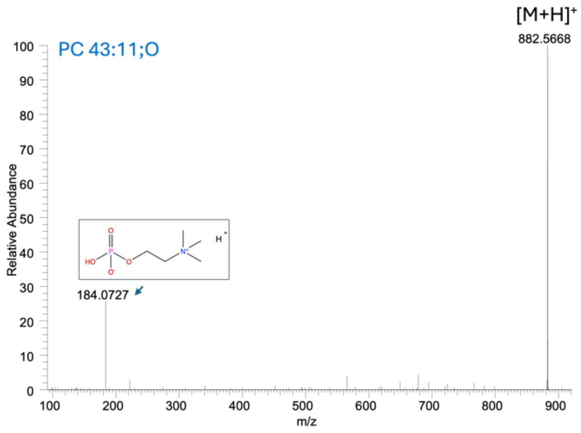
PC 43:11;O
[M+H]+
882.5668
184.0727
Relative Abundance
m/z

B

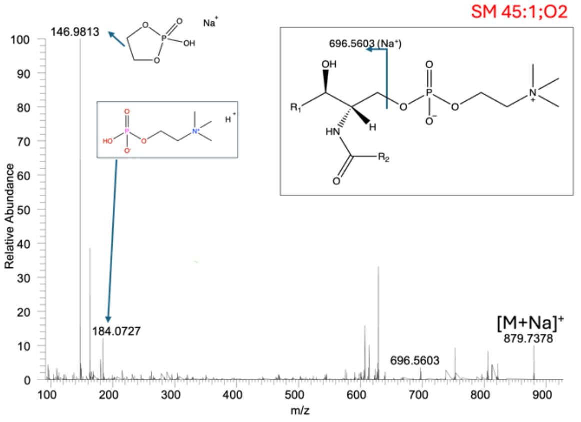
SM 45:1;O2
146.9813
696.5603 (Na+)
184.0727
696.5603
[M+Na]+
879.7378
Relative Abundance
m/z

C

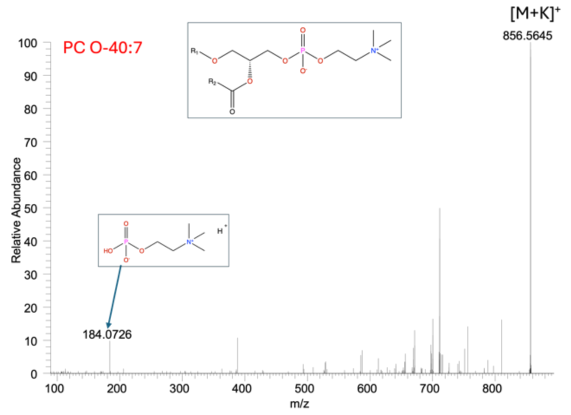
PC O-40:7
[M+K]+
856.5645
184.0726
Relative Abundance
m/z

D

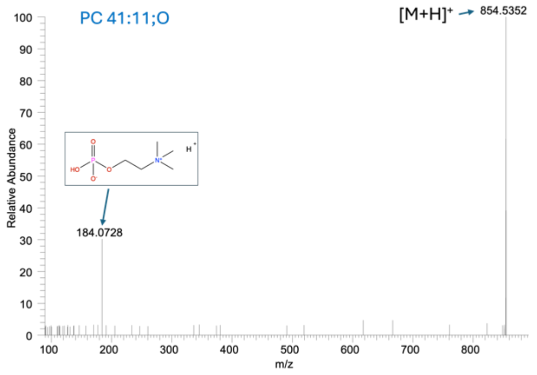
PC 41:11;O
[M+H]+
854.5352
184.0728
Relative Abundance
m/z

E

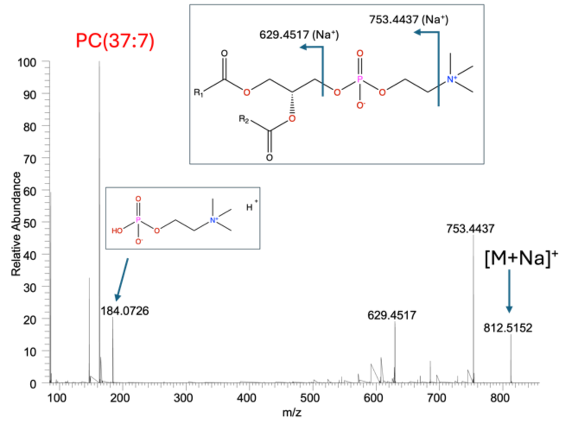
PC(37:7)
629.4517 (Na+)
753.4437 (Na+)
184.0726
753.4437
629.4517
[M+Na]+
812.5152
Relative Abundance
m/z

F

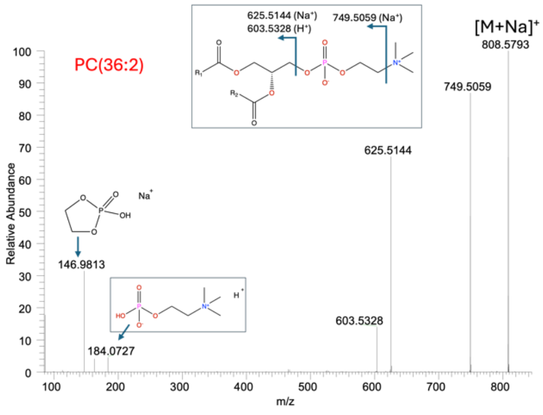
PC(36:2)
625.5144 (Na+)
603.5328 (H+)
749.5059 (Na+)
[M+Na]+
808.5793
749.5059
625.5144
146.9813
603.5328
184.0727
Relative Abundance
m/z

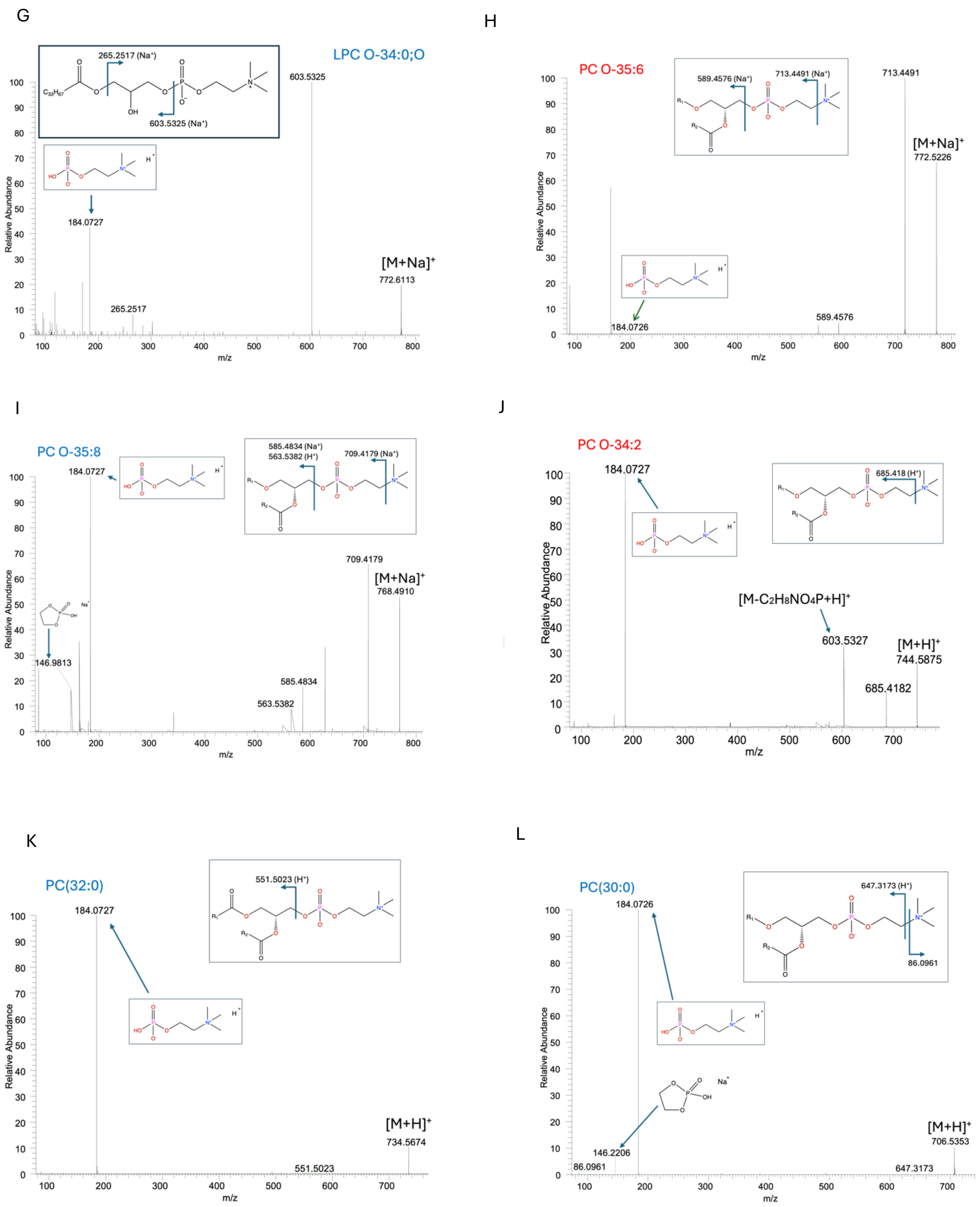
G
LPC O-34:0;O
265.2517 (Na⁺)
603.5325 (Na⁺)
184.0727
603.5325
265.2517
[M+Na]⁺
772.6113
H
PC O-35:6
589.4576 (Na⁺)
713.4491 (Na⁺)
713.4491
[M+Na]⁺
772.5226
184.0726
589.4576
I
PC O-35:8
585.4834 (Na⁺)
563.5382 (H⁺)
709.4179 (Na⁺)
184.0727
709.4179
[M+Na]⁺
768.4910
146.9813
585.4834
563.5382
J
PC O-34:2
184.0727
685.418 (H⁺)
[M-C2H8NO4P+H]⁺
603.5327
[M+H]⁺
744.5875
685.4182
K
PC(32:0)
551.5023 (H⁺)
184.0727
[M+H]⁺
734.5674
551.5023
L
PC(30:0)
647.3173 (H⁺)
86.0961
184.0726
146.2206
86.0961
[M+H]⁺
706.5353
647.3173
Relative Abundance
m/z

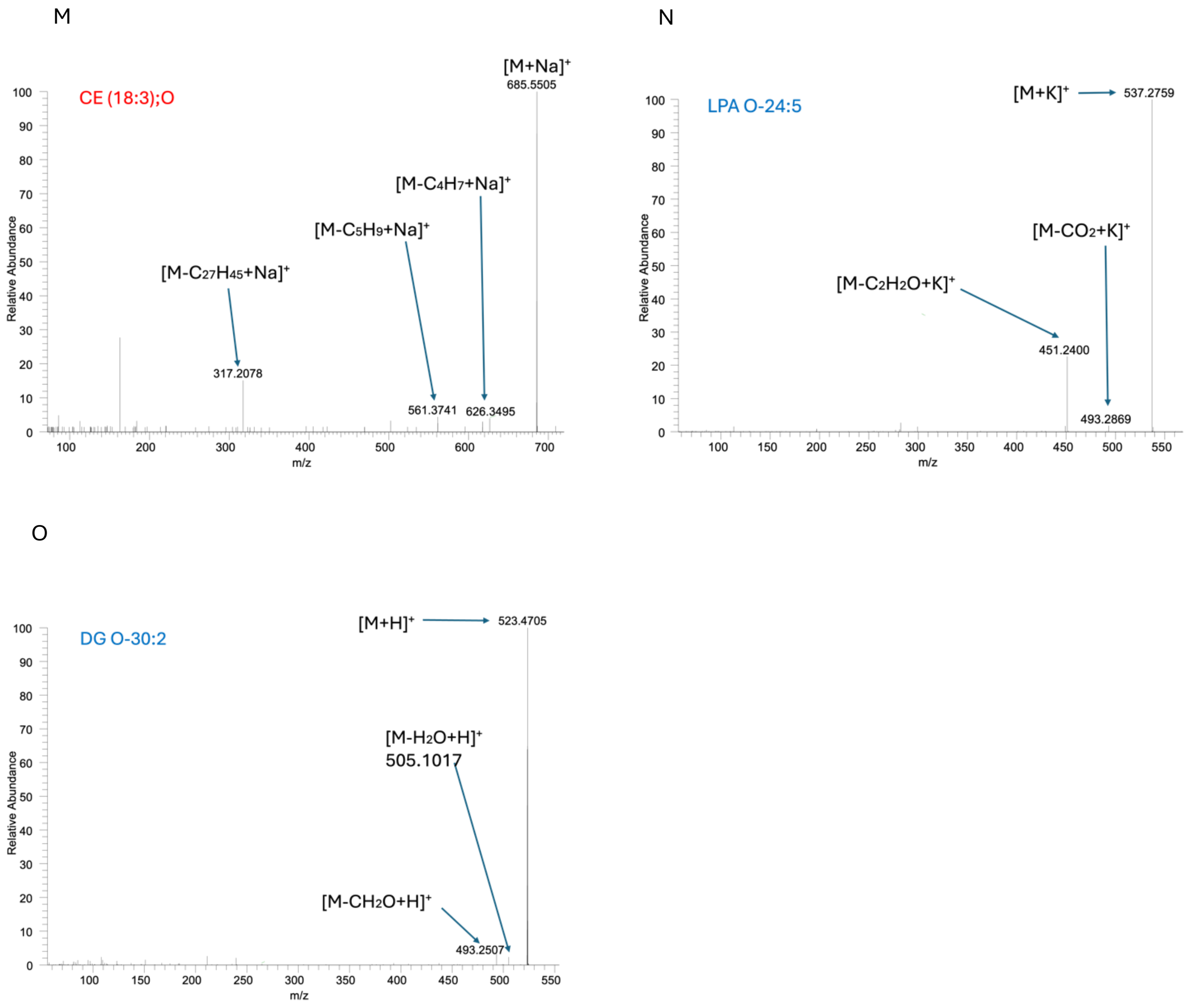

Figure S4. MS2 identification of metabolites in the positive ion mode at the single-cell level (cells in Group 4). MS/MS analyses of single cells were performed using HCD mode with a flow rate of 150 nL/min, a mass resolution of 120K (at m/z 200), and an ionization voltage of 2.9 kV for positive mode. The acquisition included one microscan and a maximum injection time of 100 ms. (A) PC 43:11;O (HCD=10), (B) SM 45:1;O2 (HCD=30), (C) PC O-40:7 (HCD=10), (D) PC 41:11;O (HCD=10), (E) PC 37:7 (HCD=25), (F) PC 36:2 (HCD=20), (G) LPC O-34:0(HCD=25), (H) PC O-35:6 (HCD=20), (I) PC O-35:8 (HCD=20), (J) PC O-34:2 (HCD=18), (K) PC 32:0 (HCD=20), (L) PC 30:0 (HCD=20), (M) CE (18:3);O (HCD=20), (N) LPA O-24:5 (HCD=25) and (O) DG O-30:2 (HCD=15). (SM: sphingomylins; PC: phosphatidylcholine; LPC: lyso phosphatidylcholine; LPA: lysophosphatidic acid; DG: diglycerides CE: cholesteryl esters).

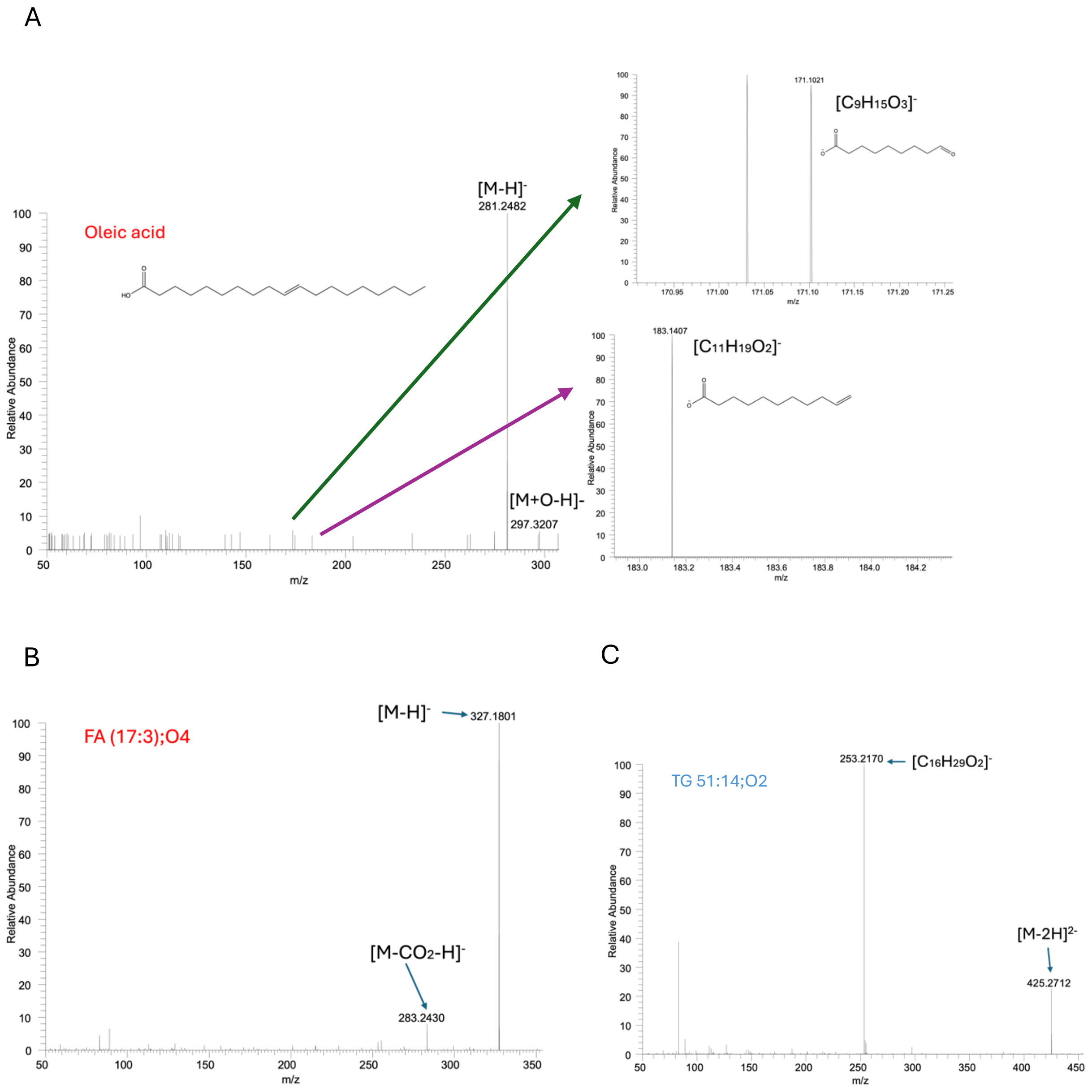

Figure S5. MS2 identification of metabolite in Group 4 cells using the Single-probe SCMS technique in negative mode. MS/MS analyses of single cells were performed using HCD mode with a flow rate of 150 nL/min, a mass resolution of 120K (at m/z 200), and an ionization voltage of −2.1 kV for negative mode. The acquisition included one microscan and a maximum injection time of 100 ms. (A) Oleic acid (HCD=35), (B) FA 17:3;O4 (HCD=20), and (C) TG 51:14;O2 (HCD=25). (FA: fatty acids; TG: triglycerides).

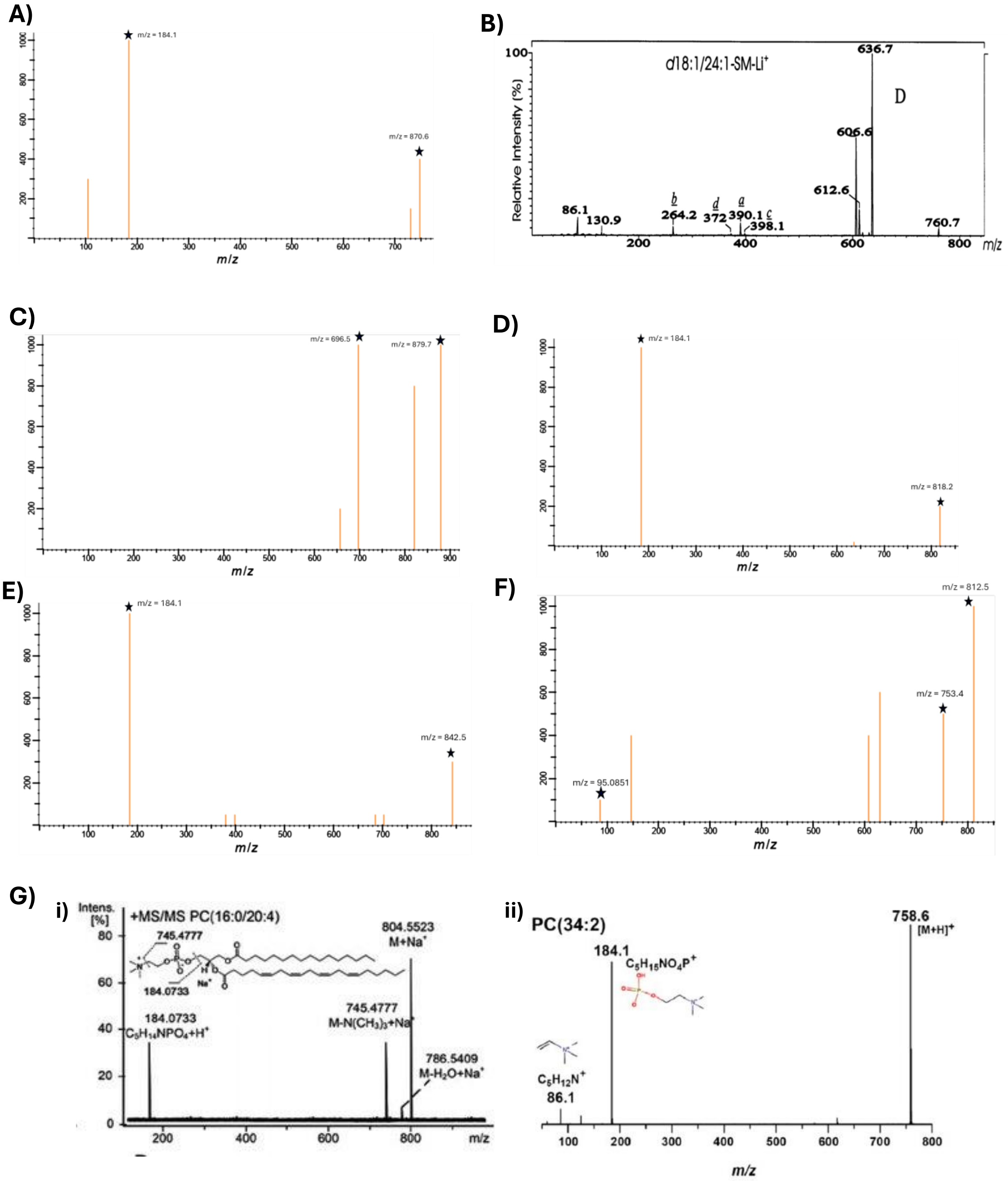
A)
m/z = 184.1
m/z = 870.6
m/z
B)
d18:1/24:1-SM-Li+
Relative Intensity (%)
636.7
D
606.6
612.6
86.1
130.9
264.2
372
390.1
398.1
760.7
m/z
C)
m/z = 696.5
m/z = 879.7
m/z
D)
m/z = 184.1
m/z = 818.2
m/z
E)
m/z = 184.1
m/z = 842.5
m/z
F)
m/z = 812.5
m/z = 753.4
m/z = 95.0851
m/z
G)
i)
Intens. [%]
+MS/MS PC(16:0/20:4)
745.4777
184.0733
804.5523
M+Na+
184.0733
C5H14NPO4+H+
745.4777
M-N(CH3)3+Na+
786.5409
M-H2O+Na+
m/z
ii)
PC(34:2)
184.1
C5H15NO4P+
758.6
[M+H]+
C5H12N+
86.1
m/z

H)

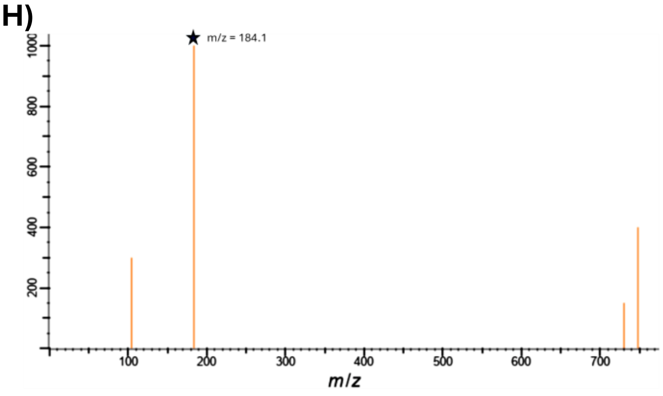
m/z = 184.1
m/z

I)

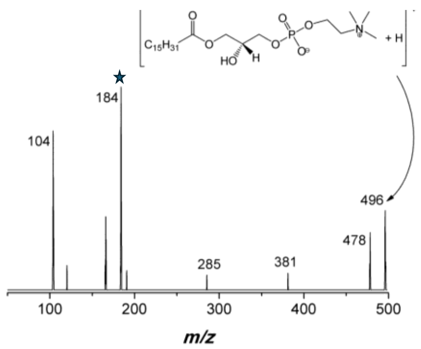
C15H31
HO
H
+ H
184
104
496
478
285
381
m/z

J)

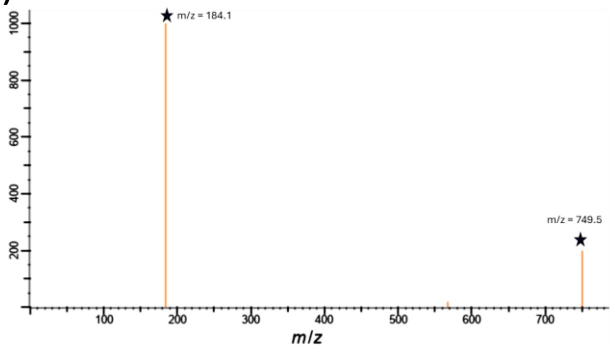
m/z = 184.1
m/z = 749.5
m/z

K)

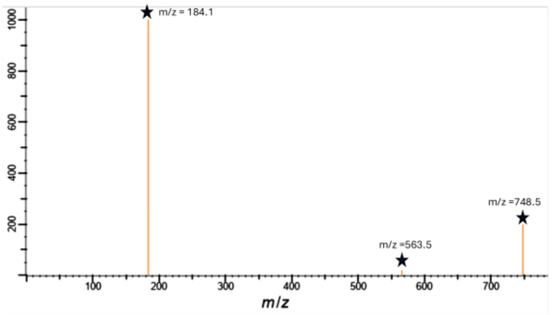
m/z = 184.1
m/z =563.5
m/z =748.5
m/z

L)

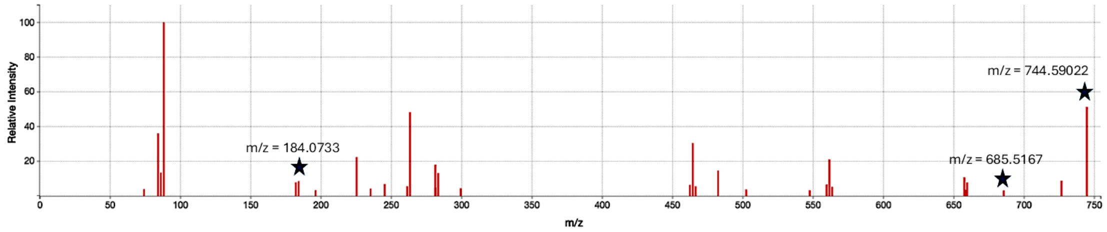
Relative Intensity
m/z = 184.0733
m/z = 744.59022
m/z = 685.5167
m/z

M)

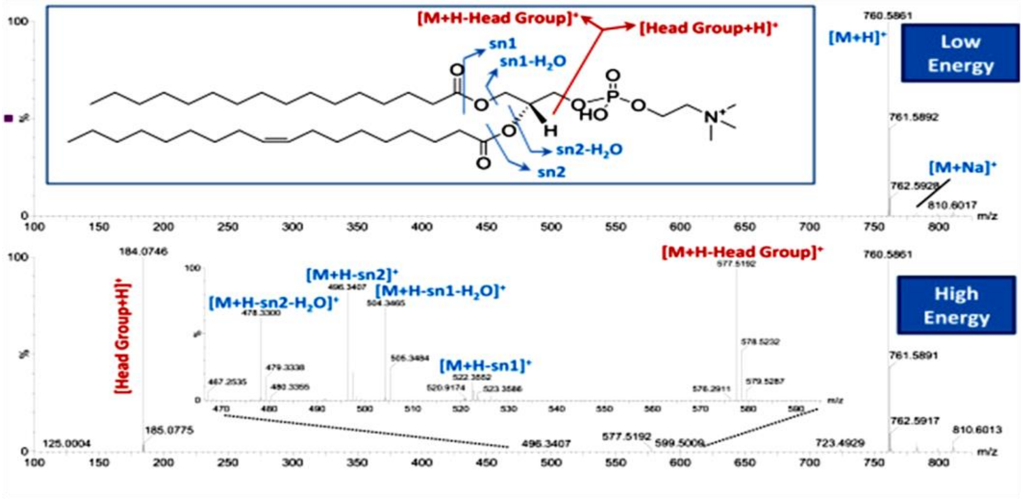
[M+H-Head Group]+
[Head Group+H]+
sn1
sn1-H2O
sn2-H2O
sn2
[M+H]+
760.5861
761.5892
[M+Na]+
762.5928
810.6017
Low Energy
184.0746
[Head Group+H]+
185.0775
125.0004
[M+H-sn2]+
[M+H-sn2-H2O]+
[M+H-sn1-H2O]+
[M+H-sn1]+
[M+H-Head Group]+
577.5192
496.3407
599.5009
723.4929
761.5891
762.5917
810.6013
High Energy
m/z

N)

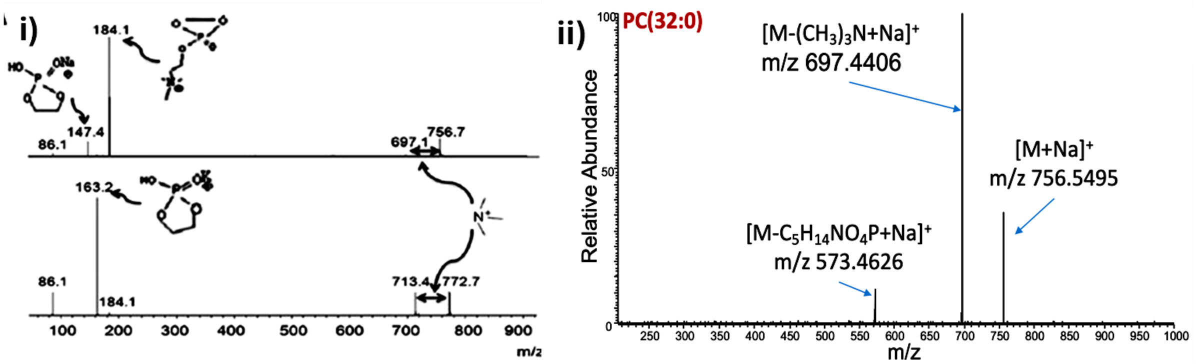

i)
184.1
147.4
86.1
697.1
756.7
163.2
86.1
184.1
713.4
772.7
100 200 300 400 500 600 700 800 900
m/z
ii)
PC(32:0)
[M-(CH3)3N+Na]+
m/z 697.4406
[M+Na]+
m/z 756.5495
[M-C5H14NO4P+Na]+
m/z 573.4626
Relative Abundance
m/z

O)

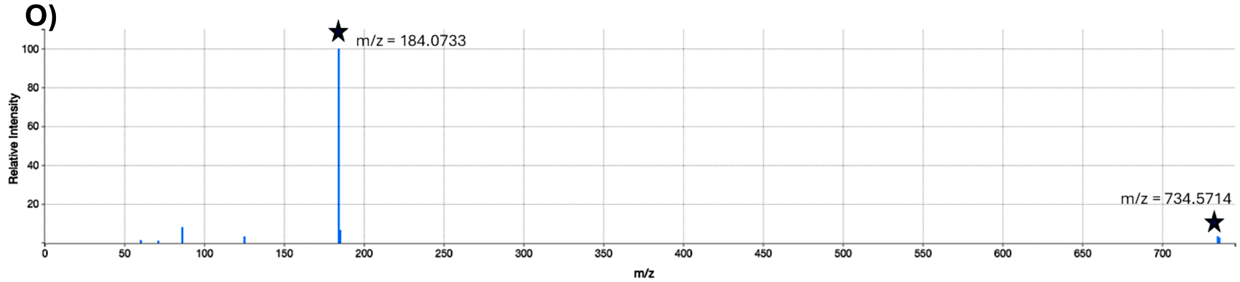

m/z = 184.0733
m/z = 734.5714
Relative Intensity
m/z

P)

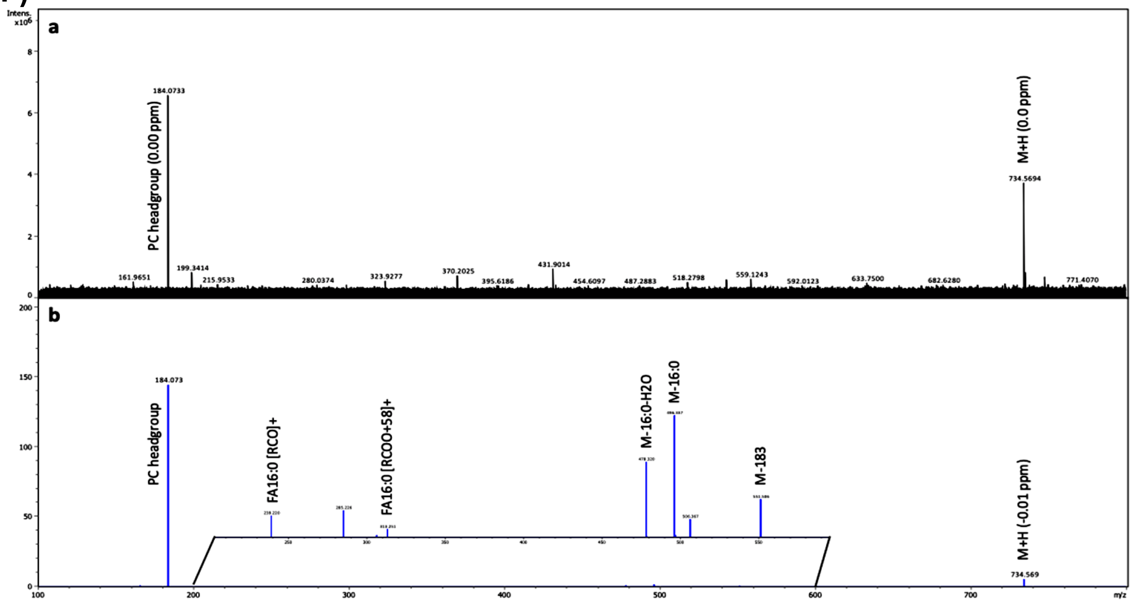

Intens.
x10^6
a
184.0733
PC headgroup (0.00 ppm)
M+H (0.0 ppm)
734.5694
161.9651
199.3414
215.9533
280.0374
323.9277
370.2025
395.6186
431.9014
454.6097
487.2883
518.2798
559.1243
592.0123
633.7500
682.6280
771.4070
b
184.073
PC headgroup
FA16:0 [RCO]+
FA16:0 [RCOO+58]+
M-16:0-H2O
M-16:0
M-183
M+H (-0.01 ppm)
734.569
m/z

Q)

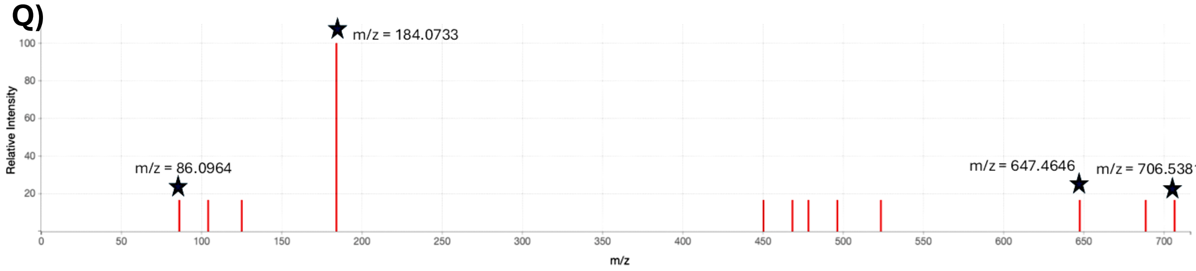

m/z = 184.0733
m/z = 86.0964
m/z = 647.4646
m/z = 706.5381
Relative Intensity
m/z

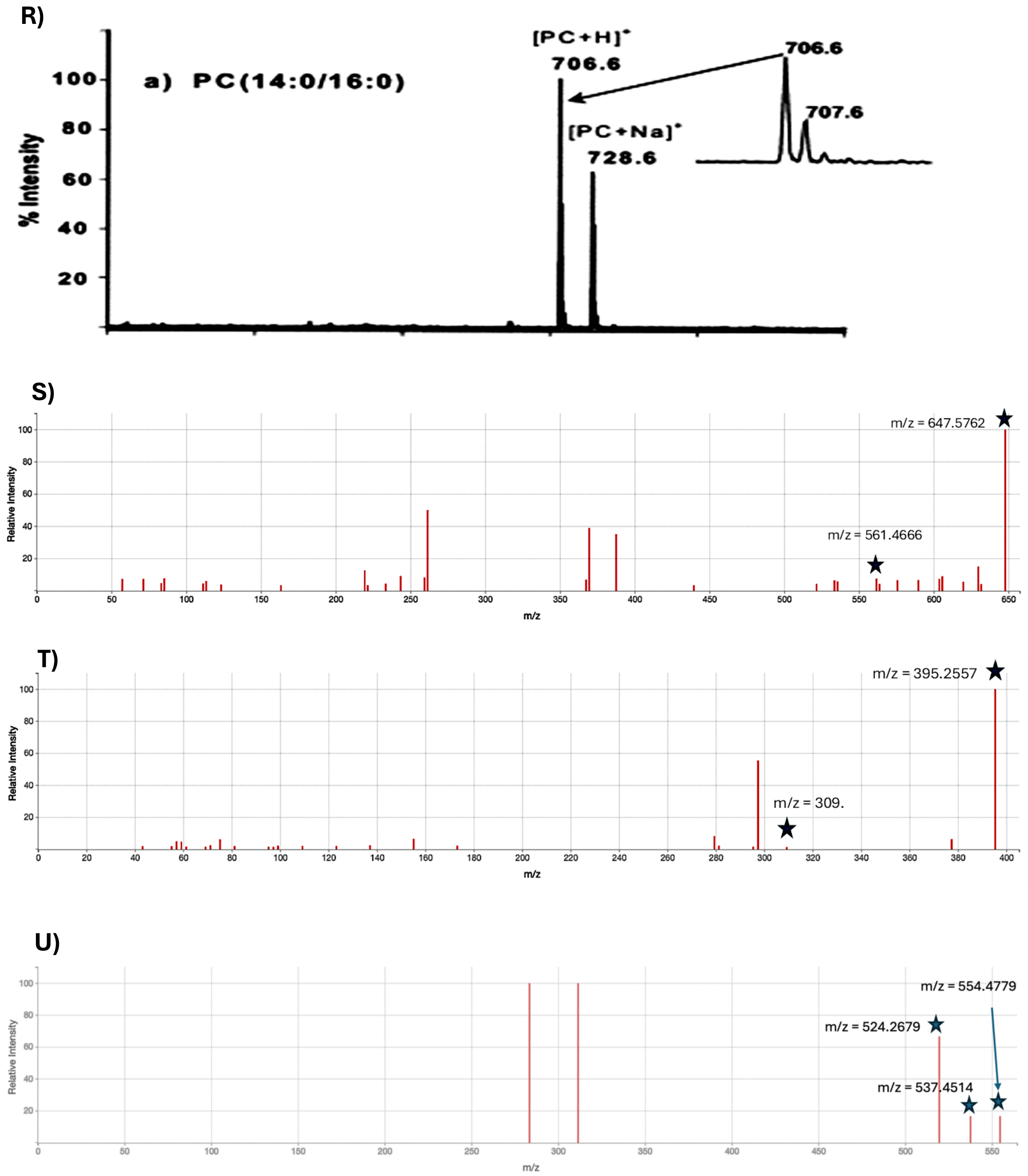

Figure S6. MS^2 spectra from previously reported studies and in silico in positive mode. (A) In silico MS^2 spectrum of PC 43:9 $[M+H]^+$ [1], highlighting the identified characteristic PC head group fragment at m/z 184.1. [Copyright © 2025 John Wiley & Sons, Inc. All Rights Reserved.] (B) Reported MS^2 spectrum of SM 45:2 $[M+Li]^+$ [2] [Reproduced with the permission Fong-Fu Hsu, John Turk. Structural determination of sphingomyelin by tandem mass spectrometry with electrospray ionization. Journal of the American Society for Mass Spectrometry. Copyright © 2000 American Society for Mass Spectrometry. Published by the American Chemical Society. All rights reserved.] and (C) in silico MS^2 spectrum of SM 45:1;O2 $[M+Na]^+$ [1]. The spectra exhibit corresponding peaks associated with the cation-generated by sphingosine and fatty acid fragments following head group cleavage. [Copyright © 2025 John Wiley & Sons, Inc. All Rights Reserved.] (D) In silico MS^2 spectrum of PC O-40:7 $[M+H]^+$ [1], showcasing the characteristic PC head group fragment detected at m/z 184.1. [Copyright © 2025 John Wiley & Sons, Inc. All Rights Reserved.] (E) In silico MS^2 spectrum of PC 41:9 $[M+H]^+$ [1], highlighting the distinctive PC

head group fragment observed at m/z 184.1. [Copyright © 2025 John Wiley & Sons, Inc. All Rights Reserved.] (F) In silico MS^2 spectrum of PC 37:7 $[M+Na]^+$ [1], showcasing the distinctive PC head group fragment observed at m/z 184.1, fragment at m/z 753.4 resulting from amine group cleavage. [Copyright © 2025 John Wiley & Sons, Inc. All Rights Reserved.] (G) Reported MS^2 spectrum of (i) PC 36:4 $[M+Na]^+$ [3] [Reproduced from Ref. 3 with permission from Yanbo Wei, Yangyang Zhang, Yu Lin, Lin Li, Jian'an Liu, Zhenpeng Wang,b Shaoxiang Xiong and Zhenwen Zhao from the Royal Society of Chemistry. A uniform 2,5-dihydroxybenzoic acid layer as a matrix for MALDI-FTICR MS-based lipidomics.] and (ii) PC 34:2 $[M+H]^+$ [4] [Reproduced with the Hyun Jun Jang, J. H. P., Ga Seul Lee, Sung Bae Lee, Jeong Hee Moon, Joon Sig Choi ,Tae Geol Lee, Sohee Yoon. Comparison of Lipid Profiles in Head and Brain Samples of DrosophilaMelanogaster Using Electrospray Ionization Mass Spectrometry (ESI-MS). Mass Spectrometry Letters. Copyright ©Korean Society for Mass Spectrometry. Published by the Mass Spectrometry Letters. All rights reserved.], showcasing the distinctive PC head group fragment observed at m/z 184.1, fragment at m/z 745.4777 resulting from amine group cleavage which is in a good agreement with the m/z 749.5059 in PC 36:2 $[M+Na]^+$. (H) In silico MS^2 spectrum of LPC 34:0 $[M+H]^+$ [1] [Copyright © 2025 John Wiley & Sons, Inc. All Rights Reserved.] and (I) Reported MS^2 spectrum of LPC 34:0 [5]. The spectra exhibit PC head group fragment observed at m/z 184.1. [Reproduced with the permission Enzo Cadoni, Petr Vanhara, Elisa Valletta, Elisabetta Pinna, Sarah Vascellari, Graziano Caddeo, Francesco Isaia, Alessandra Pani, Josef Havel, Tiziana Pivetta. Mass spectrometric discrimination of phospholipid patterns in cisplatin-resistant and -sensitive cancer cells. Rapid Communications in Mass Spectrometry © 2018 John Wiley & Sons, Ltd.] (J) In silico MS^2 spectrum of PC O-35:6 $[M+H]^+$ [1], highlighting the identified characteristic PC head group fragment at m/z 184.1 and parent ion. [Copyright © 2025 John Wiley & Sons, Inc. All Rights Reserved.] (K) In silico MS^2 spectrum of PC O-35:7 $[M+H]^+$, highlighting the identified characteristic PC head group fragment at m/z 184.1, fragment at m/z 563.5 resulting from PC head group cleavage which is in a good agreement with the m/z 563.5382 in PC O-35:8 $[M+Na]^+$ and parent ion. [Copyright © 2025 John Wiley & Sons, Inc. All Rights Reserved.] (L) In silico MS^2 spectrum of PC O-34:2 $[M+H]^+$ [6] and (M) Reported MS^2 spectrum of PC 34:1, highlighting the identified characteristic PC head group fragment at m/z 184.0733, fragment at m/z 685.5167 resulting from amine group cleavage and parent ion[7]. [Reproduced with the permission Yolanda Chico, Beatriz Abad-García, Begoña Ochoa, María José Martínez. Lipidomic data uncover extensive heterogeneity in phosphatidylcholine structural variants in HepG2 cells. Data in Brief © 2019 The Author(s). Published by Elsevier Inc.] (N) (i) [8] [Reproduced with the permission Selina Rahman Shanta,Chang Soon Choi,Jeong Hwa Lee,Chan Young Shin,Young Jun Kim,Kyun-Hwan Kim,Kwang Pyo Kim. Global changes in phospholipids identified by MALDI MS in rats with focal cerebral ischemia[S]. © 2012 ASBMB. Currently published by Elsevier Inc; originally published by American Society for Biochemistry and Molecular Biology] , (ii) [9] [Reproduced with the permission Xingxiu Chen,Mei Sun,Zhibo Yang. Single cell mass spectrometry analysis of drug-resistant cancer cells: Metabolomics studies of synergetic effect of combinational treatment. Analytica Chimica Acta. © 2022 Elsevier B.V. All rights reserved.] , Reported MS^2 spectrum of PC 32:0 $[M+Na]^+$, (O) In silico MS^2 spectrum of PC 32:0 $[M+H]^+$ [6], (P) Reported MS^2 spectrum of PC 32:0 $[M+H]^+$ highlighting the identified characteristic PC head group fragment at m/z 184.0733 and parent ion[10]. (Q) In silico MS^2 spectrum of PC 30:0 $[M+H]^+$ [6] and (R) Reported MS^2 spectrum of PC 30:0[10] highlighting the identified characteristic PC head group fragment at m/z 184.0733, amine fragment m/z 86.0964, fragment at m/z 647.4646 resulting from PC head group cleavage and parent ion. [Reproduced with the permission Khalid A Al-Saad,William F Siems,H.H Hill,Vladimir Zabrouskov,N.Richard Knowles. Structural analysis of phosphatidylcholines by post-source decay matrix-assisted laser desorption/ionization time-of-flight mass spectrometry. Journal of the American Society for Mass Spectrometry. Copyright © 2003 American Society for Mass Spectrometry. Published by the American Chemical Society. All rights reserved.] (S) In silico MS^2 spectrum of CE 18:3 $[M+H]^+$ [6], highlighting the identified fragment at m/z 561.4666 resulting from cleavage of alky group which is in a good agreement with the m/z 561.3741 in CE 18:3;O. (T) In silico MS^2 spectrum of LPA (P-16:0) [6], highlighting the identified fragment at m/z 309 resulting from cleavage of C2H2O group which is in a good agreement with the m/z 451.2400 in LPA O-24:5. (U) In silico MS^2 spectrum of DG 30:2 $[M+NH4]^+$ [6] highlighting the identified fragment at m/z 537.4514 resulted from cleavage of water, m/z 524.2679 resulted from cleavage of CH2O is in good agreement with m/z 505.1017, m/z 493.2507 in DG O-30:2 $[M+NH4]^+$ respectively.

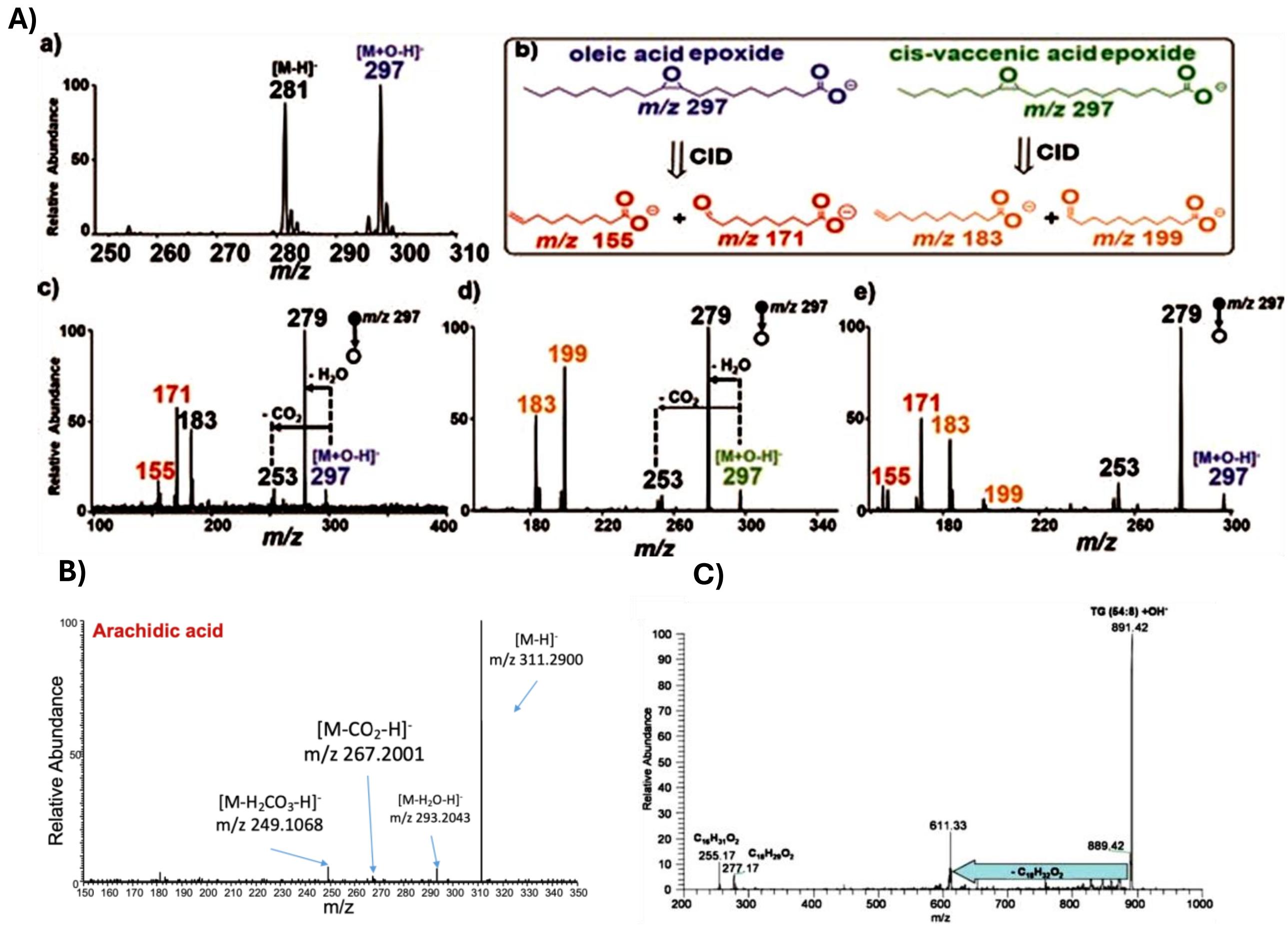

Figure S7. MS^2 spectra from previously reported studies and in silico in negative mode. (A) Reported MS^2 spectra of Oleic acid[11] with identified m/z 281, 297,171 and 183. [*Reproduced from Ref. 11 with permission K. Chintalapudi and A. K. Badu-Tawiah from the Royal Society of Chemistry. An integrated electrocatalytic nESI-MS platform for quantification of fatty acid isomers directly from untreated biofluids.]* (B) Reported MS^2 spectrum of Arachidic acid[9] showcasing the fragment observed at m/z 267.2001, resulting from CO2 group cleavage which is in a good agreement with the m/z 283.2430 in FA 17:3;O4. [*Reproduced with the permission Xingxiu Chen,Mei Sun,Zhibo Yang. Single cell mass spectrometry analysis of drug-resistant cancer cells: Metabolomics studies of synergetic effect of combinational treatment. Analytica Chimica Acta. © 2022 Elsevier B.V. All rights reserved.]* (C) Reported MS^2 spectrum of TG (54:8) $[M\text{-}OH]^{-}$ [11] showcasing the fragment observed at m/z 611.33, resulting from acyl group cleavage which is in a good agreement with the m/z 253.2170 in TG 51:14;O2. [*Reproduced with the permission Stefanie Gerbig, Zoltán Takáts. Analysis of triglycerides in food items by desorption electrospray ionization mass spectrometry. Rapid Commun Mass Spectrom. Copyright (c) 2010 John Wiley & Sons, Ltd.]*

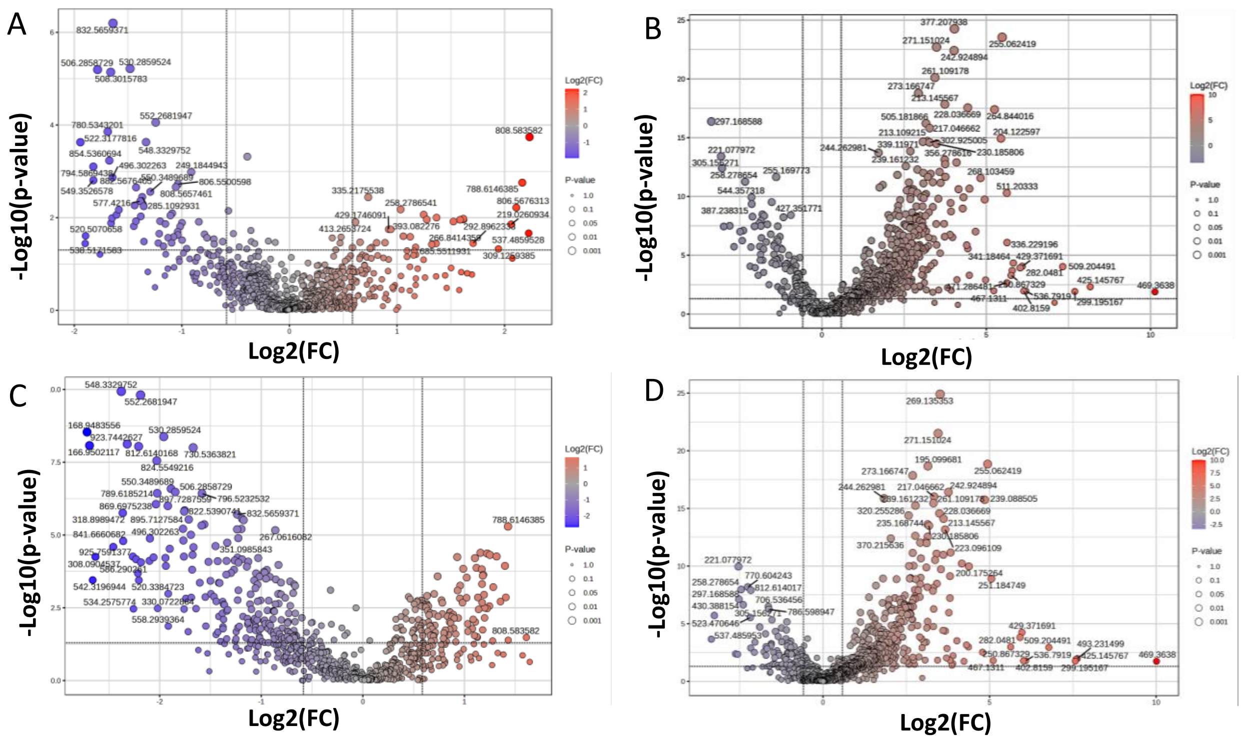

Figure S8. Volcano plots illustrating significantly changed species (fold change > 1.5 and p-value < 0.05) in the positive ion mode through pairwise comparison. **(A)** Group 1 vs. Group 2 (22 increased and 38 decreased metabolites), **(B)** Group 3 vs. Group 4 (324 increased and 53 decreased metabolites). **(C)** Group 1 vs. Group 3 (58 increased and 134 decreased metabolites). **(D)** Group 2 vs. Group 4 (144 increased and 100 decreased metabolites).

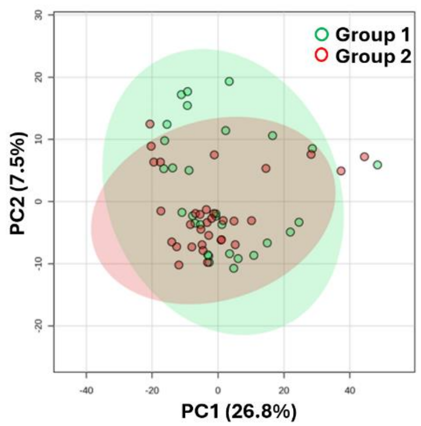

Figure S9. PCA of SCMS results obtained from HEK 293T cells in Group 1 and Group 2 in the positive ion mode. HEK 293T cells were prepared using the same procedures as those for HCT-116 cells in Group 1 and Group 2.

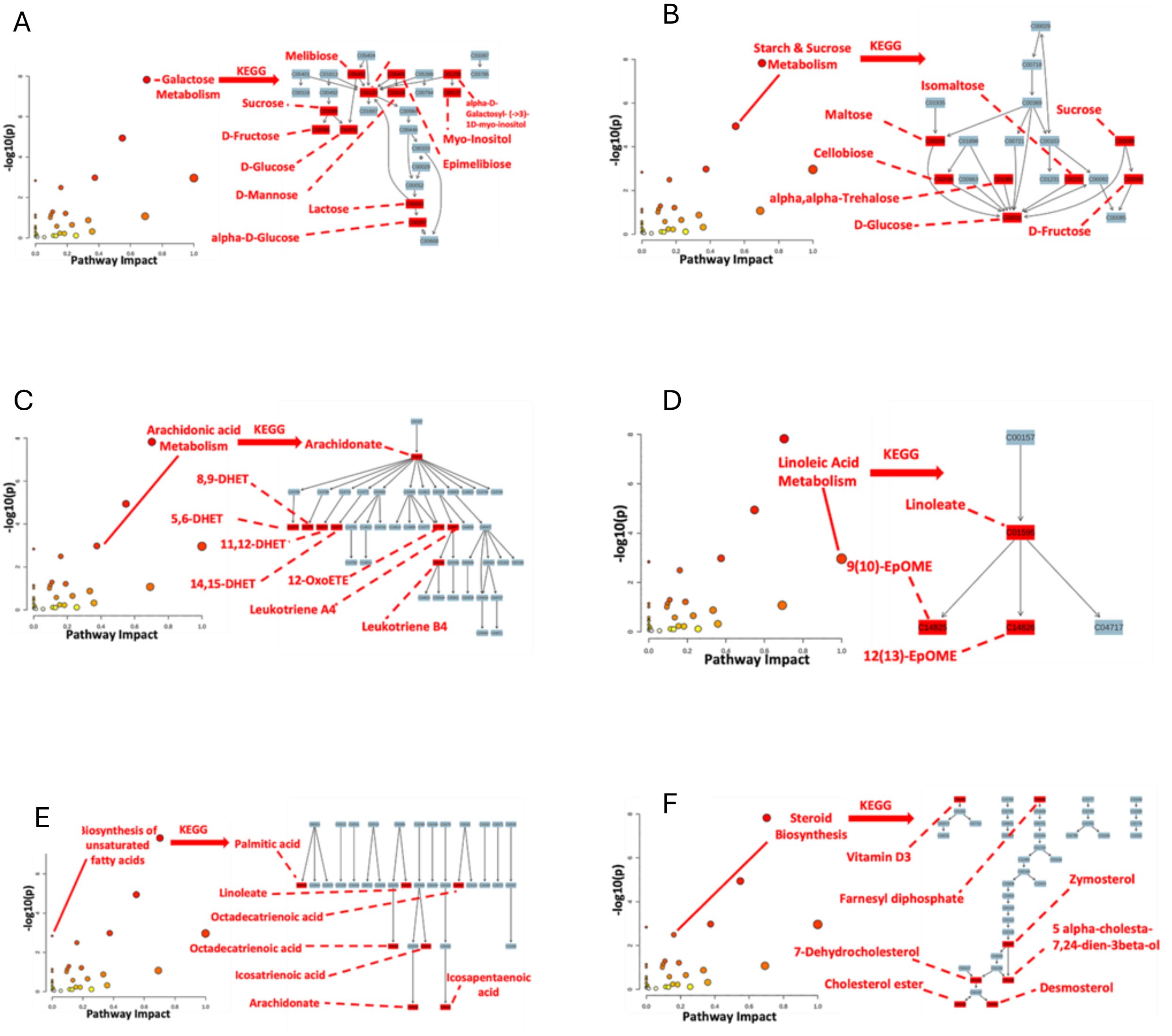

Figure S10. Pathway analysis using significantly changed metabolites ($p<0.05$ and FC > 1.5) from the comparison between Group 3 and Group 4 in the positive ion mode. **(A)** Galactose metabolism (FDR = 1.19E-6). **(B)** Starch and sucrose metabolism (FDR = 4.58E-4). **(C)** Arachidonic acid metabolism (FDR = 0.0218). **(D)** Linoleic acid metabolism (FDR = 0.0218). **(E)** Biosynthesis of unsaturated fatty acids pathway (FDR = 0.0233). **(F)** Steroid biosynthesis pathway (FDR = 0.0427). Identified (based on MS/MS) and tentatively labeled (based on comparison of accurate m/z and database) metabolites are shown in red font.

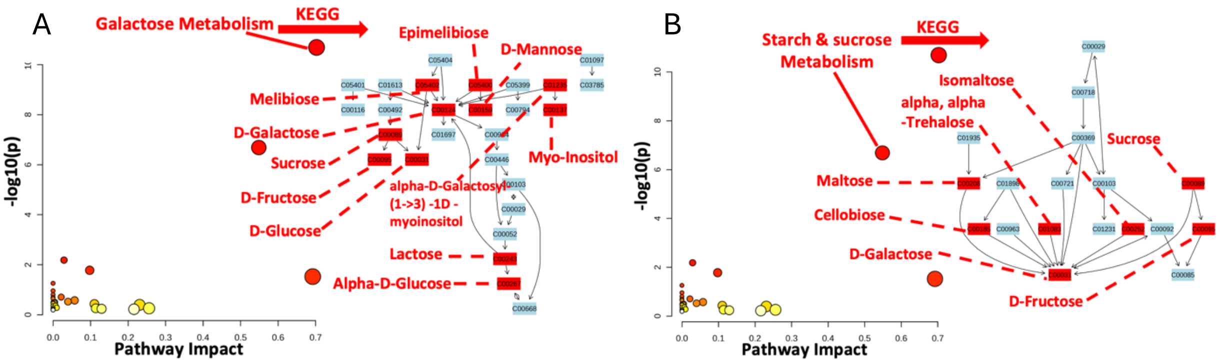

Figure S11. Pathway analysis using significantly changed metabolites (p<0.05 and FC > 1.5) from the comparison between Group 2 and Group 4 in the positive ion mode. **(A)** Galactose metabolism (FDR = 1.63 x 10^{-9}). **(B)** Starch and sucrose metabolism (FDR = = 8.24 x 10^{-6}). Identified (based on MS/MS) and tentatively labeled (based on comparison of accurate m/z and database) metabolites are shown in red font.

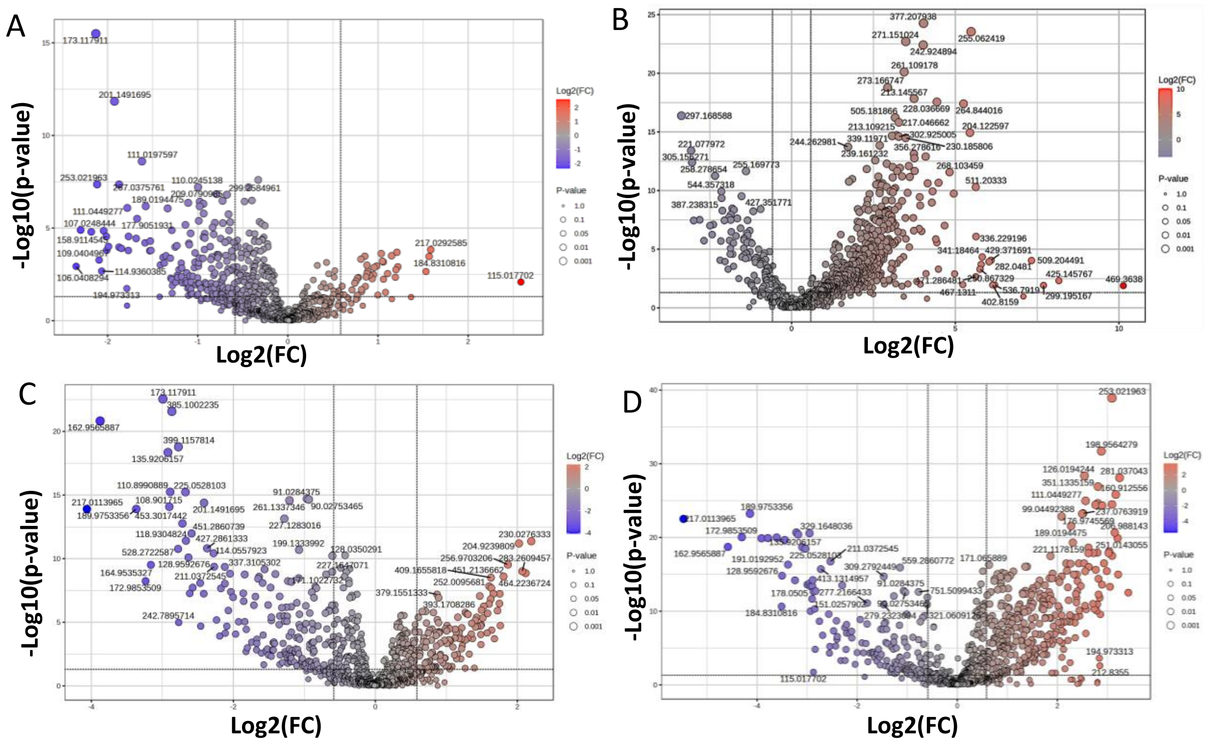

Figure S12. Volcano plots illustrating significantly changed species (fold change > 1.5 and p-value < 0.05) in the negative ion mode through pairwise comparison. **(A)** Group 1 vs. Group 2 (20 increased and 125 decreased metabolites). **(B)** Group 3 vs. Group 4 (235 increased and 56 decreased). **(C)** Group 1 vs. Group 3 (86 increased and 141 decreased metabolites). **(D)** Group 2 vs. Group 4 (87 increased and 187 decreased metabolites).

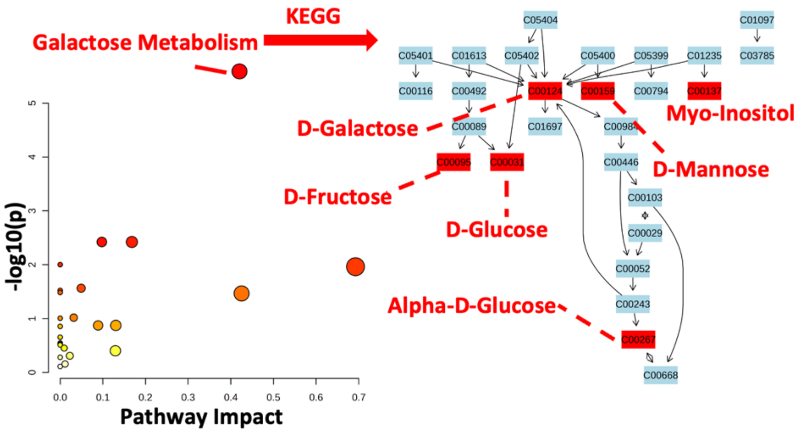

Figure S13. Pathway analysis using significantly changed metabolites (p<0.05 and FC > 1.5) from the comparison between Group 1 and Group 2 in the negative ion mode. Pathway analysis revealed that galactose metabolism (FDR = 2.06 x 10-4) significantly changed. Identified (based on MS/MS) and tentatively labeled (based on comparison of accurate m/z and database) metabolites are shown in red font.

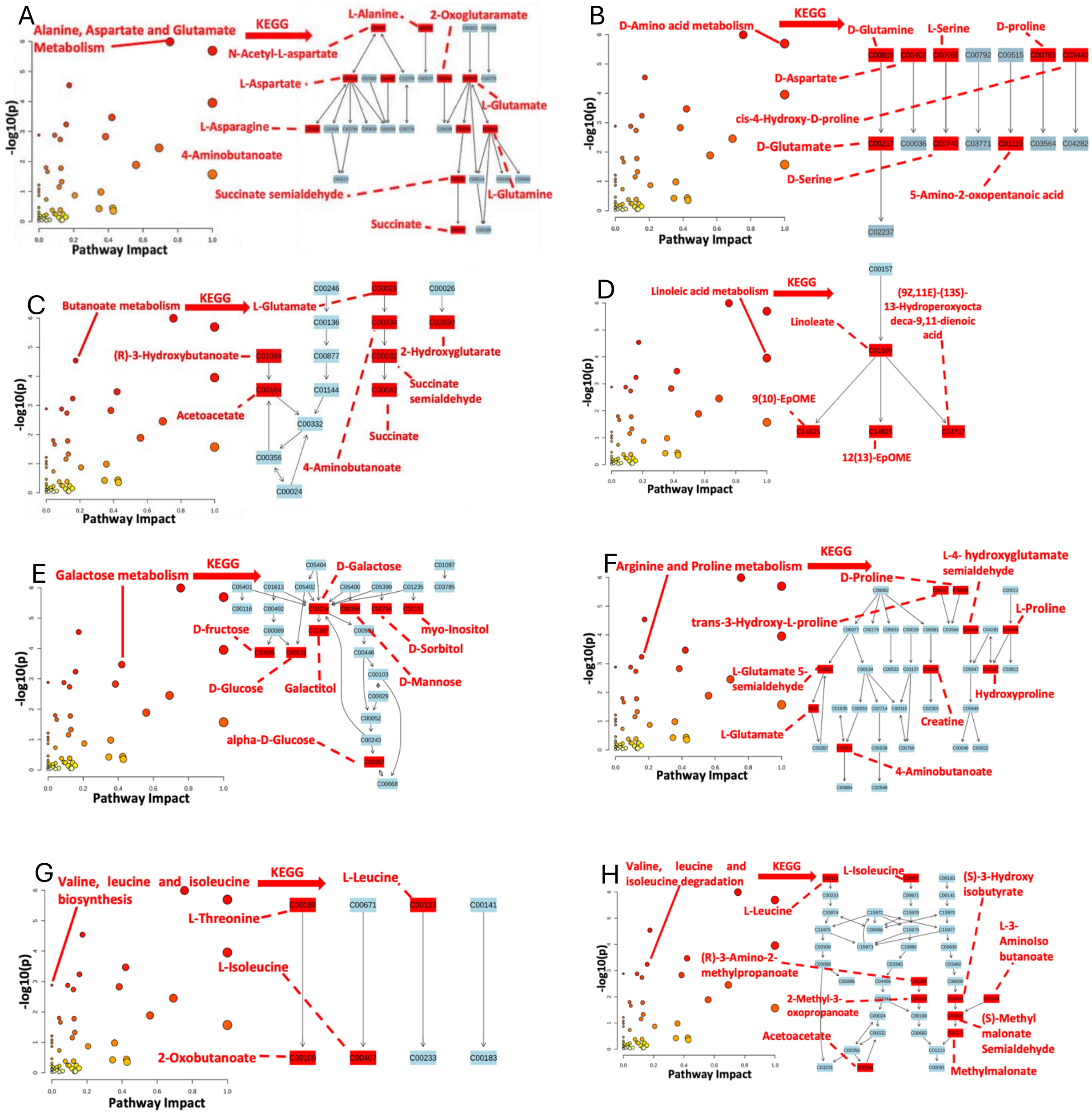
A
Alanine, Aspartate and Glutamate Metabolism
KEGG
L-Alanine
2-Oxoglutaramate
N-Acetyl-L-aspartate
L-Aspartate
L-Glutamate
L-Asparagine
4-Aminobutanoate
Succinate semialdehyde
L-Glutamine
Succinate
-log10(p)
Pathway Impact
B
D-Amino acid metabolism
KEGG
D-Glutamine
L-Serine
D-proline
C00819
C00402
C00065
C00792
C00515
C00763
C03440
D-Aspartate
cis-4-Hydroxy-D-proline
D-Glutamate
C00217
C00036
C00740
C03771
C01110
C03564
C04282
D-Serine
5-Amino-2-oxopentanoic acid
C02237
-log10(p)
Pathway Impact
C
Butanoate metabolism
KEGG
L-Glutamate
C00246
C00025
C00026
C00136
C00334
C02630
(R)-3-Hydroxybutanoate
C01089
C00877
C00232
2-Hydroxyglutarate
C00164
C01144
C00042
Succinate semialdehyde
Acetoacetate
C00332
Succinate
C00356
4-Aminobutanoate
C00024
-log10(p)
Pathway Impact
D
Linoleic acid metabolism
KEGG
C00157
(9Z,11E)-(13S)-13-Hydroperoxyocta deca-9,11-dienoic acid
Linoleate
C01595
9(10)-EpOME
C14825
C14826
C04717
12(13)-EpOME
-log10(p)
Pathway Impact
E
Galactose metabolism
KEGG
C05404
D-Galactose
C01097
C05401
C01613
C05402
C05400
C05399
C01235
C03785
C00116
C00492
C00124
C00159
C00794
C00137
D-fructose
C00089
C01697
myo-Inositol
D-Sorbitol
D-Glucose
Galactitol
D-Mannose
C00446
C00103
C00029
C00052
alpha-D-Glucose
C00243
C00668
-log10(p)
Pathway Impact
F
Arginine and Proline metabolism
KEGG
D-Proline
L-4- hydroxyglutamate semialdehyde
trans-3-Hydroxy-L-proline
L-Proline
L-Glutamate 5-semialdehyde
Hydroxyproline
Creatine
L-Glutamate
4-Aminobutanoate
-log10(p)
Pathway Impact
G
Valine, leucine and isoleucine biosynthesis
KEGG
L-Leucine
L-Threonine
C00188
C00671
C00123
C00141
L-Isoleucine
2-Oxobutanoate
C00109
C00407
C00233
C00183
-log10(p)
Pathway Impact
H
Valine, leucine and isoleucine degradation
KEGG
L-Isoleucine
(S)-3-Hydroxy isobutyrate
L-Leucine
L-3-AminoIso butanoate
(R)-3-Amino-2-methylpropanoate
2-Methyl-3-oxopropanoate
(S)-Methyl malonate Semialdehyde
Acetoacetate
Methylmalonate
-log10(p)
Pathway Impact

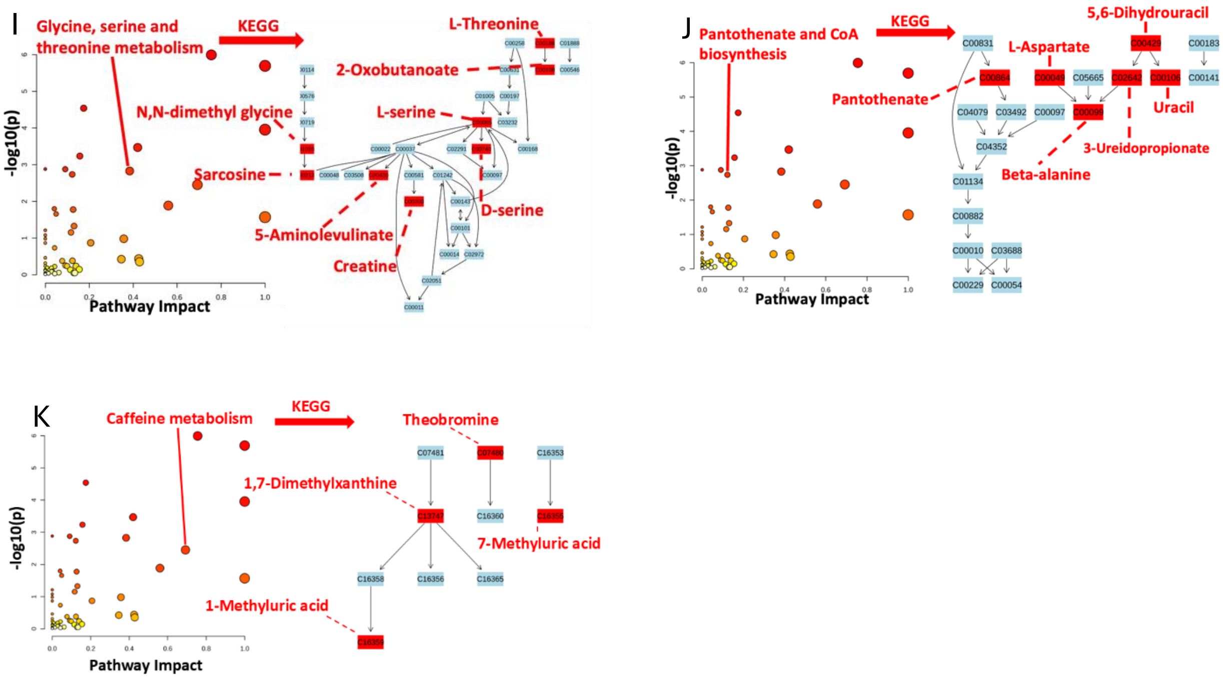

Figure S14. Pathway analysis using significantly changed metabolites ($p<0.05$ and $FC > 1.5$) from the comparison between Group 3 and Group 4 in the negative ion mode. **(A)** Alanine, aspartate, and glutamate metabolism, showing a highly significant association (FDR = 8.09E-05). **(B)** D-amino acid metabolism (FDR = 8.09E-05). **(C)** Butanoate metabolism pathway (FDR = 0.000768). **(D)** linoleic acid metabolism pathway (FDR = 2.22 E-03). **(E) G**alactose metabolism (FDR = 0.0054311). **(F)** Arginine and proline metabolism (FDR = 0.0077486). **(G)** Biosynthesis of valine, leucine, and isoleucine (FDR = 0.013182). **(H)** Degradation pathways of valine, leucine, and isoleucine (FDR = 0.013182). **(I)** Metabolism of glycine, serine, and threonine (FDR = 0.013182). **(J)** Biosynthesis of pantothenate and coenzyme A (CoA) (FDR = 0.014686). **(K)** Caffeine metabolism (FDR = 0.002563). Identified (based on MS/MS) and tentatively labeled (based on comparison of accurate m/z and database) metabolites are shown in red font.

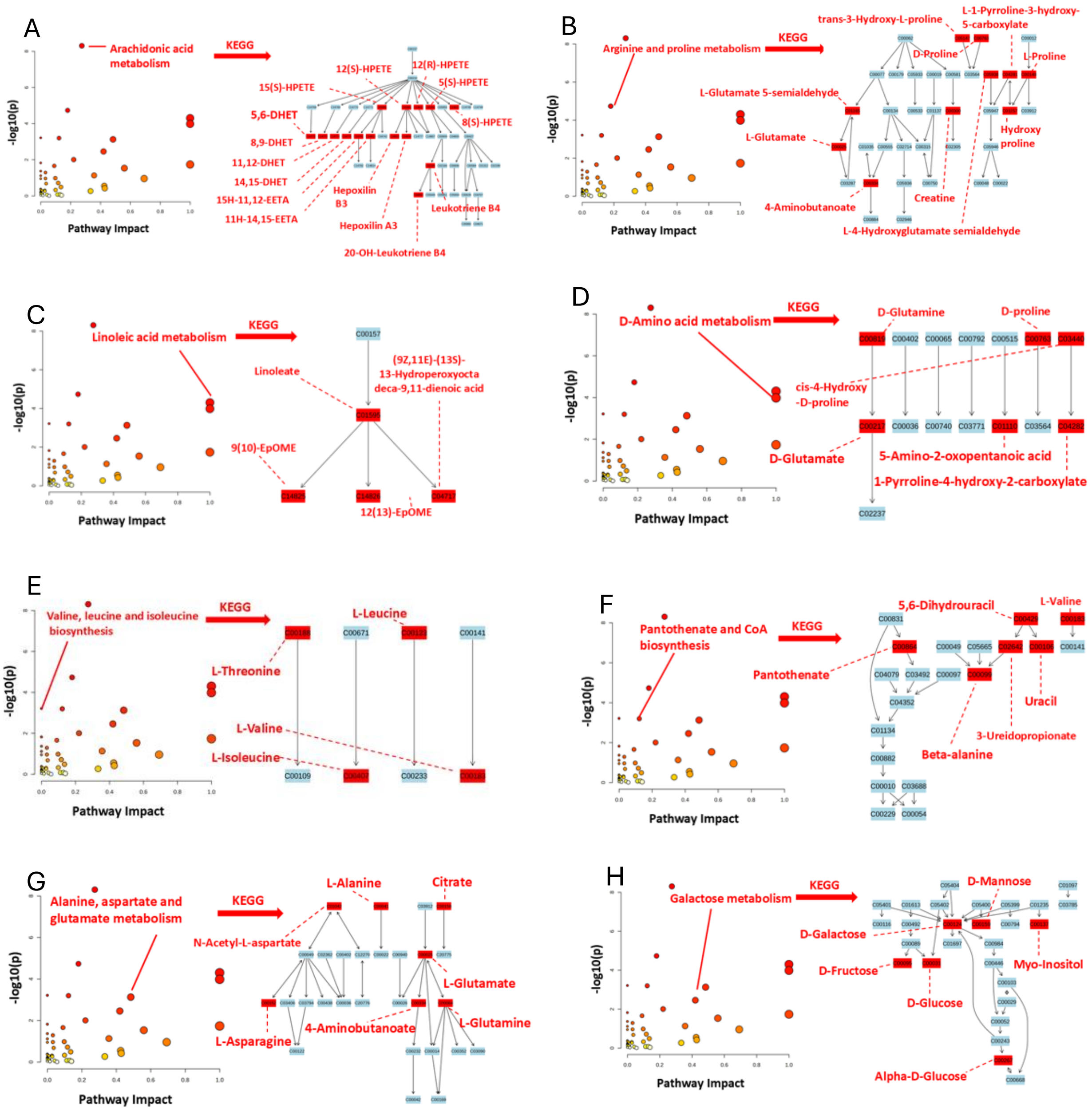

Figure S15. Pathway analysis using significantly changed metabolites (p<0.05 and FC > 1.5) from the comparison between Group 1 and Group 3 in the negative ion mode. **(A)** Arachidonic acid metabolism (FDR = 3.96 x 10^{-7}). **(B)** Arginine and proline metabolism (FDR = 7.40 x 10^{-4}). **(C)** Linoleic acid metabolism (FDR = 1.33 x 10^{-3}). **(D)** D-Amino acid metabolism (FDR = 2.07 x 10^{-3}). **(E)** Valine, leucine and isoleucine biosynthesis (FDR = 8.45 x 10^{-3}). **(F)** Pantothenate and CoA biosynthesis (FDR = 8.45x 10^{-3}). **(G)** Alanine, aspartate and glutamate metabolism (FDR = 8.51x 10^{-3}). **(H)** Galactose metabolism (FDR = 3.48 x 10^{-2}). Identified (based on MS/MS) and tentatively labeled (based on comparison of accurate m/z and database) metabolites are shown in red font.

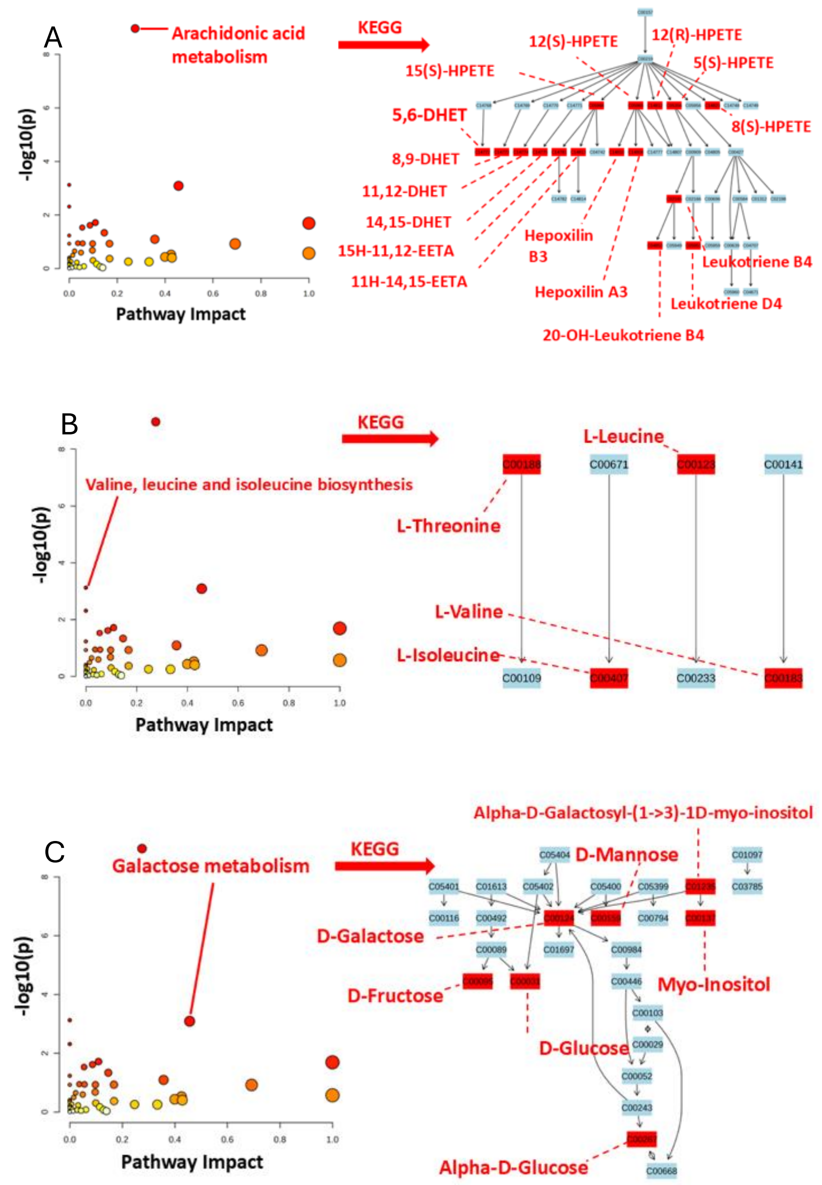

Figure S16. Pathway analysis using significantly changed metabolites ($p<0.05$ and FC > 1.5) from the comparison between Group 2 and Group 4 in the negative ion mode. **(A)** Arachidonic acid metabolism (FDR = 8.63 x10^{-8}). **(B)** Valine, leucine and isoleucine biosynthesis (FDR = 2.17 x10^{-2}). **(C)** Galactose metabolism (FDR = 2.17 x10^{-2}). Identified (based on MS/MS) and tentatively labeled (based on comparison of accurate m/z and database) metabolites are shown in red font.

Post hoc power analysis

Post hoc power analysis was performed to determine if adequate single cells were analyzed in our studies. Because LN_2 quenching and freeze-drying are the key steps to preserve cells' metabolites, it is necessary to investigate the effect of omitting both steps. In addition, sample storage in -80 °C freezer is commonly used in laboratory studies, so it is necessary to consider its influence on cell metabolites. Thus, it is reasonable to conduct *post hoc* power analysis of SCMS results of cells in Groups 3 vs 4 (Table SI_1 and Table SI_2 (xlsx)). In fact, among all paired comparisons, Groups 3 vs 4 resulted in the largest number of significantly altered metabolites and pathways in both positive and negative ion modes (Tables S4 and S8). Using ions that were significantly altered, our results indicate that, in general, analyzing 20-25 cells in each group is adequate (power > 0.8) to differentiate these two groups.

References

(1) Tsugawa, H.; Ikeda, K.; Takahashi, M.; Satoh, A.; Mori, Y.; Uchino, H.; Okahashi, N.; Yamada, Y.; Tada, I.; Bonini, P.; et al. A lipidome atlas in MS-DIAL 4. *Nat Biotechnol* **2020**, *38* (10), 1159-1163. DOI: 10.1038/s41587-020-0531-2.

(2) Fong-Fu Hsu, J. T. Structural Determination of Sphingomyelin by Tandem Mass Spectrometry With Electrospray Ionization. *American Society for Mass Spectrometry* **2000**, *11*, 13.

(3) Wei, Y.; Zhang, Y.; Lin, Y.; Li, L.; Liu, J.; Wang, Z.; Xiong, S.; Zhao, Z. A uniform 2,5-dihydroxybenzoic acid layer as a matrix for MALDI-FTICR MS-based lipidomics. *Analyst* **2015**, *140* (4), 1298-1305. DOI: 10.1039/c4an01964d .

(4) Hyun Jun Jang, J. H. P., Ga Seul Lee, Sung Bae Lee, Jeong Hee Moon, Joon Sig Choi ,Tae Geol Lee, Sohee Yoon. Comparison of Lipid Profiles in Head and Brain Samples of DrosophilaMelanogaster Using Electrospray Ionization Mass Spectrometry (ESI-MS). *Mass Spectrometry Letters* **2019**, *10*, 7. DOI: 10.5478/MSL.2019.10.1.11.

(5) Cadoni, E.; Vanhara, P.; Valletta, E.; Pinna, E.; Vascellari, S.; Caddeo, G.; Isaia, F.; Pani, A.; Havel, J.; Pivetta, T. Mass spectrometric discrimination of phospholipid patterns in cisplatin-resistant and -sensitive cancer cells. *Rapid Commun Mass Spectrom* **2019**, *33* (1), 97-106. DOI: 10.1002/rcm.8320 .

(6) Wishart, D. S.; Guo, A.; Oler, E.; Wang, F.; Anjum, A.; Peters, H.; Dizon, R.; Sayeeda, Z.; Tian, S.; Lee, B. L.; et al. HMDB 5.0: the Human Metabolome Database for 2022. *Nucleic Acids Res* **2022**, *50* (D1), D622-D631. DOI: 10.1093/nar/gkab1062 .

(7) Chico, Y.; Abad-Garcia, B.; Ochoa, B.; Martinez, M. J. Lipidomic data uncover extensive heterogeneity in phosphatidylcholine structural variants in HepG2 cells. *Data Brief* **2019**, *27*, 104608. DOI: 10.1016/j.dib.2019.104608 .

(8) Shanta, S. R.; Choi, C. S.; Lee, J. H.; Shin, C. Y.; Kim, Y. J.; Kim, K. H.; Kim, K. P. Global changes in phospholipids identified by MALDI MS in rats with focal cerebral ischemia. *J Lipid Res* **2012**, *53* (9), 1823-1831. DOI: 10.1194/jlr.M022558.

(9) Chen, X.; Sun, M.; Yang, Z. Single cell mass spectrometry analysis of drug-resistant cancer cells: Metabolomics studies of synergetic effect of combinational treatment. *Anal Chim Acta* **2022**, *1201*, 339621. DOI: 10.1016/j.aca.2022.339621.

(10) Al-Saad, K. A.; Siems, W. F.; Hill, H. H.; Zabrouskov, V.; Knowles, N. R. Structural analysis of phosphatidylcholines by post-source decay matrix-assisted laser desorption/ionization time-of-flight mass spectrometry. *J Am Soc Mass Spectrom* **2003**, *14* (4), 373-382. DOI: 10.1016/S1044-0305(03)00068-0.

(11) Chintalapudi, K.; Badu-Tawiah, A. K. An integrated electrocatalytic nESI-MS platform for quantification of fatty acid isomers directly from untreated biofluids. *Chem Sci* **2020**, *11* (36), 9891-9897. DOI: 10.1039/d0sc03403g.
